# Targeting programmed cell death in diabetic kidney disease: from molecular mechanisms to pharmacotherapy

**DOI:** 10.1186/s10020-024-01020-5

**Published:** 2024-12-20

**Authors:** Fengzhao Liu, Zhenyu Yang, Jixin Li, Tao Wu, Xiangyu Li, Lijuan Zhao, Wenru Wang, Wenfei Yu, Guangheng Zhang, Yunsheng Xu

**Affiliations:** 1 First College of Clinical Medicine, Shandong University of Traditional Chinese Medicine, Jinan, 250014 China; 2 Graduate School of Heilongjiang University of Chinese Medicine, Harbin, 150040 China; 3 Xi Yuan Hospital, China Academy of Chinese Medical Sciences, Beijing, 100091 China; 4 School of Traditional Chinese Medicine, Beijing University of Chinese Medicine, Beijing, 100029 China; 5 Wangjing Hospital, China Academy of Chinese Medical Sciences, Beijing, 100102 China; 6 Department of Endocrinology, Second Affiliated Hospital of Shandong University of Traditional Chinese Medicine, Jinan, 250001 China

**Keywords:** Diabetic kidney disease, Programmed cell death, Natural products, Podocytes, Tubular epithelial cells

## Abstract

Diabetic kidney disease (DKD), one of the most prevalent microvascular complications of diabetes, arises from dysregulated glucose and lipid metabolism induced by hyperglycemia, resulting in the deterioration of renal cells such as podocytes and tubular epithelial cells. Programmed cell death (PCD), comprising apoptosis, autophagy, ferroptosis, pyroptosis, and necroptosis, represents a spectrum of cell demise processes intricately governed by genetic mechanisms in vivo. Under physiological conditions, PCD facilitates the turnover of cellular populations and serves as a protective mechanism to eliminate impaired podocytes or tubular epithelial cells, thereby preserving renal tissue homeostasis amidst hyperglycemic stress. However, existing research predominantly elucidates individual modes of cell death, neglecting the intricate interplay and mutual modulation observed among various forms of PCD. In this comprehensive review, we delineate the diverse regulatory mechanisms governing PCD and elucidate the intricate crosstalk dynamics among distinct PCD pathways. Furthermore, we review recent advancements in understanding the pathogenesis of PCD and explore their implications in DKD. Additionally, we explore the potential of natural products derived primarily from botanical sources as therapeutic agents, highlighting their multifaceted effects on modulating PCD crosstalk, thereby proposing novel strategies for DKD treatment.

## Introduction

Diabetic kidney disease (DKD) stands as one of the most prevalent microvascular complications of diabetes mellitus (DM), characterized by chronic kidney injury induced by prolonged hyperglycemia, and stands as a predominant contributor to end-stage renal disease (ESRD) (Jung and Yoo 2022). The hallmark characteristics of DKD include the onset of proteinuria and the progressive decline in glomerular filtration rate (GFR). Presently, clinical management options remain limited, primarily focusing on symptomatic interventions such as hypoglycemia and antihypertensive therapies, while advanced preventive and therapeutic measures are lacking (Gnudi et al. 2016; McGrath and Edi 2019). Despite the intricate pathogenesis of DKD, encompassing oxidative stress, and immuno-inflammation, extensive research signifies that renal cell demise, particularly of podocytes and tubular cells, intimately correlates not only with the aforementioned processes, influencing DKD progression but also directly impacts renal-associated tissues and structures, thereby driving DKD development (Erekat 2022; McGrath and Edi 2019; Pang et al. 2024; Yang et al. 2023a). Hence, investigating cell death regulation emerges as a novel pivotal approach for DKD prevention and management, with programmed cell death (PCD) constituting a cornerstone thereof.

Cell death, an irreversible phenomenon in biological systems, maintains bodily homeostasis under normal conditions, yet exacerbates tissue damage and disease progression under pathological circumstances. Cell demise dichotomizes into necrosis and PCD, with the former representing a chaotic passive demise following robust physicochemical or biological stimuli, while the latter denotes an active, orderly cell death instigated by gene modulation upon encountering internal or external environmental cues, serving as a self-defense mechanism to eliminate unwanted or abnormal cells (Kowalski et al. 2023; Kulkarni and Hardwick 2023). PCD encompasses apoptosis, autophagy, ferroptosis, pyroptosis, and necroptosis, each exhibiting distinct characteristics and morphological alterations (Kulkarni and Hardwick 2023). The first two forms lack cell rupture, content extravasation, or inflammatory responses, hence termed 'silent' PCD, while pyroptosis and necroptosis induce cell rupture and provoke inflammatory responses, and ferroptosis, garnering recent attention, represents a novel form of PCD (Song et al. 2023). PCD homeostasis significantly impacts human body growth and development, with its imbalance fostering excessive cell lysis or accumulation of deleterious substances, thus impeding normal cellular functions and jeopardizing internal environment stability, culminating in diverse ailments (Fei et al. 2024; Song et al. 2023; Zhao et al. 2023b). Consequently, persistent hyperglycemia in DKD patients engenders PCD imbalance, thereby disrupting the physiological activity of pertinent renal cells like podocytes and renal tubular epithelial cells (RTECs), thus fueling DKD progression (Erekat 2022).

Each kidney comprises approximately 1 million nephrons, the structural and functional units of the kidney, encompassing renal corpuscles (comprising glomeruli and renal capsules) and renal tubules. Pathological alterations, such as glomerular basement membrane (GBM) thickening, mesangial expansion, and tubular atrophy, predispose DKD patients to increased proteinuria and diminished GFR (Hu et al. 2023). Consensus delineates podocyte pathology as pivotal in DKD progression. As terminally differentiated glomerular epithelial cells devoid of replicative capacity, podocytes represent the final barrier to glomerular filtration, lacking cell replacement post-injury until glomerulosclerosis ensues (Li et al. 2023b). Conversely, RTECs, the predominant renal tubular cell type, pivotal in renal reabsorption, are particularly sensitive to internal and external stimuli owing to their substantial energy demands. Increasingly, RTEC lesions are acknowledged not only in mid-to-late-stage DKD but also in early-stage disease progression (Zhou et al. 2023). Studies illustrate that early DKD stages induce high glucose (HG)-mediated inflammatory and oxidative stress stimuli, culminating in morphological podocyte and RTEC alterations such as epithelial-mesenchymal transdifferentiation (EMT), hypertrophy, and detachment (Li et al. 2023b). Concurrently, PCD maintains renal structural homeostasis by eliminating necrotic cells. Nevertheless, as DKD advances, PCD dysregulation precipitates normal cell demise. Ergo, preserving podocyte and RTEC PCD homeostasis assumes significance in DKD prevention and management.

A myriad of natural compounds (NPs) sourced from plants, animals, and microorganisms are gaining traction in novel drug development due to their accessibility, cost-effectiveness, and minimal side effects (Newman and Cragg 2016, 2020). Moreover, research elucidates NPs' multi-target, multi-pathway regulation of PCD in renal cells, pivotal in internal environment homeostasis maintenance, DKD prevention, and management. Remarkably, PCD modalities do not operate in isolation; for instance, apoptosis, pyroptosis, and necroptosis form a cohesive cell death system wherein one pathway can compensate for another. While considerable studies explore NP-regulated PCD in conditions like osteoporosis, ulcerative colitis, and diabetic cardiomyopathy, a dearth of literature reviews delineate the collective impact of diverse PCD regulation modalities and NP-mediated PCD regulation in DKD treatment (Chen et al. 2024; Li et al. 2023c; Xuan and Zhang 2023). Hence, this study elucidates the interplay between distinct PCD modalities and investigates the prospective association between various PCD forms and DKD, providing insights into NP mechanisms and efficacy in modulating PCD for DKD treatment, thereby bridging extant research gaps and highlighting NPs' potential as a therapeutic avenue for DKD treatment.

## Methods

We conducted a systematic search of the literature using PubMed, Embase, Cochrane and Web of Science databases for the period from database inception to 04/2024. We use the following terms: “Diabetic kidney disease”, "diabetic nephropathy", "programmed cell death", "apoptosis", "autophagy", " ferroptosis ", "pyroptosis", " necroptosis " and "natural products". The references of eligible studies were subjected to a manual review. Two researchers then independently conducted a search and assessment of the included studies, with any disagreements in the literature search being resolved by a third researcher through the application of consensus. A total of 311 relevant articles were obtained through a systematic literature search. Following the removal of duplicates, the titles and abstracts of the remaining 284 articles were initially read. Subsequently, reviews, case reports, letters, and non-subject-related and non-English writing were excluded, resulting in 92 documents being included in the manuscript for review. The study has been registered with the PROSPERO database (CRD42024550264).

## Apoptosis

### Overview of apoptosis

Apoptosis, also referred to as type I PCD, represents the predominant form of PCD. Sequential activation of the cysteine-aspartate protease (caspases) family in the inactive state is an important feature of apoptosis initiation. Apoptosis pathways include the intrinsic endoplasmic reticulum (ER) pathway, mitochondrial pathway, and exogenous death receptor pathway, depending on the source of apoptotic signals.

#### Apoptosis mediated by the endoplasmic reticulum pathway

Affected by the accumulation of misfolded or unfolded proteins and imbalances in Ca^2+^ within the cell, ER stress occurs, subsequently triggering the unfolded protein response (UPR) to protect the ER (Yong et al. 2021). However, excessive ER stress can activate three ER transmembrane proteins that regulate UPR: protein kinase-like ER kinase (PERK), inositol-requiring enzyme 1α (IRE1α), and activating transcription factor 6α (ATF6α), leading to apoptosis (Sundaram et al. 2018; Yong et al. 2021). Specifically, upon release, PERK phosphorylates eukaryotic translation initiation factor 2α (elF2α), which in turn induces the expression of the activating transcription factor ATF4, which promotes the expression of the apoptosis signal molecule CHOP/GADD153 to promote apoptosis (Fan and Jordan 2022). Released IRE1 collects the cytoplasmic regulatory protein TRAF-2 and activates c-jun terminal Kinase (JNK), which inhibits the activity of apoptosis-inhibiting proteins of the Bcl-2 family. Also IRE1 can enhance apoptosis by activating Caspase-12 and promoting transcriptional expression of CHOP (Huang et al. 2019). ATF6 is cleaved by Golgi to form a short-chain ATF6 and transferred to the nucleus to induce the expression of CHOP/GADD153 (Yang et al. 2023c). In addition, the outflow of Ca^2 +^ will affect the activity of mitochondria and Bcl-2 family, and can also activate neutral cysteine endopeptidase Calpain to cause caspase cascade reaction, leading to apoptosis (Song et al. 2024).

#### Apoptosis mediated by the mitochondrial pathway

Mitochondria are not only the centre of the cellular respiratory chain and oxidative phosphorylation, but also a major regulator of apoptosis. The B-cell lymphoma-2 (Bcl-2) family of proteins are the major regulators controlling the release of mitochondria-associated apoptotic factors and are classified into the anti-apoptotic proteins Bcl-2, B-cell lymphoma-extra-large (Bcl-xL), and the pro-apoptotic proteins Bcl-2-associated X protein (Bax), Brassinosteroid-insensitive 1 (BRI1)-associated kinase (Bak), Bad, Bid and Bim, etc. (King et al. 2023). Under normal conditions, Bcl-2 and Bcl-xL form heterodimers with Bax and Bak to maintain the integrity of the outer mitochondrial membrane. Upon cellular stimulation by endogenous signals such as growth factor deprivation or DNA damage, Bax expression is activated and forms oligomeric complexes with Bak that insert into the mitochondrial outer membrane, leading to a decrease in mitochondrial membrane potential (MMP) and alterations in mitochondrial membrane permeability (Czabotar and Garcia-Saez 2023; King et al. 2023). Subsequently, pro-apoptotic factors are released from the mitochondria, halting the synthesis of adenosine triphosphate (ATP) within the mitochondria, ultimately driving the cell towards apoptosis (Tait and Green 2013). Specifically, pro-apoptotic factors in mitochondria, cytochrome *C* (Cyt *C*), which is first released into the cytoplasm, can bind to apoptotic peptidase activating factor 1 (Apaf-1) to form an apoptotic complex, and then activating downstream Caspase-3/6/7/9, and cutting the relevant substrates in the cell, eventually leading to apoptosis (Bock and Tait 2020). Secondly inhibitor of apoptosis proteins (IAPs) can bind to the above apoptotic complexes and Caspase-3/6/7 while Smac/Diablo and HtrA2/Omi indirectly promote apoptosis by inhibiting the activity of IAPs (Cong et al. 2019). Finally, AIF and Endo G are transferred to the nucleus, causing chromosome condensation and DNA fragmentation in the nucleus, leading to apoptosis (Bock and Tait 2020).

#### Apoptosis mediated by the death receptor pathway

**Fig. 1:**
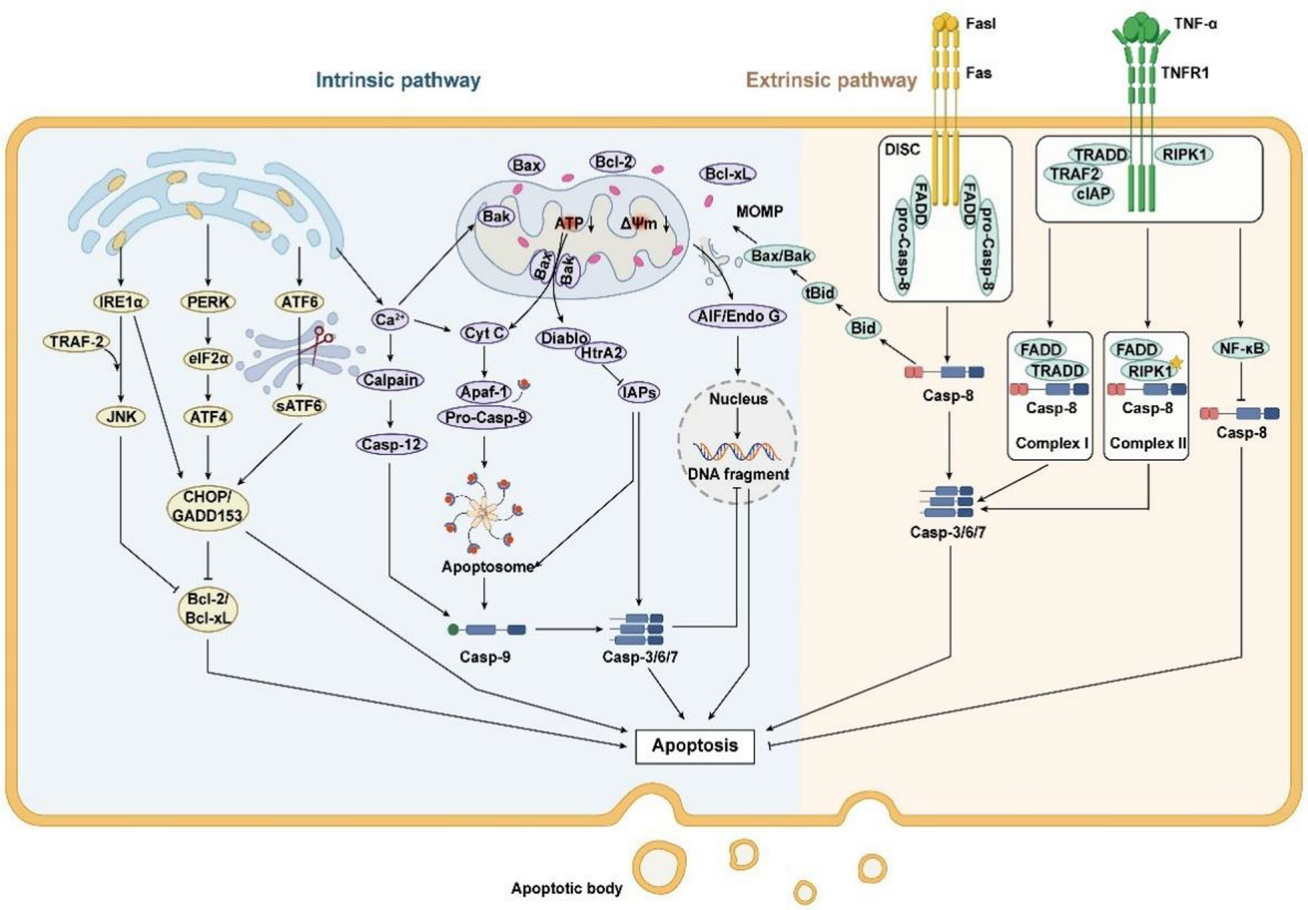
Intrinsic pathway and extrinsic pathway of apoptosis include endoplasmic reticulum stress pathway, mitochondrial pathway and death receptor pathway. The endoplasmic reticulum pathway is caused by the excessive activation of UPR indecued by ERS, including IRE1α, PERK/eIF2α/ATF4 and ATF6 pathways, causing the activation of CHOP to induce apoptosis. Mitochondrial pathway is regulated by Bcl-2 protein family. The Bax/Bak complex leads to increased permeability of the mitochondrial membrane, which releases several pro-apoptotic proteins, including Cyt *C*, and initiates the next step of the caspase-processing cascade, inducing apoptosis. Ca^2+^ in the endoplasmic reticulum pathway is not only involved in the endoplasmic reticulum stress pathway, but also affects the mitochondrial pathway by influencing the Bax complex. The death receptor pathway means that the death ligand binds to the receptor after receiving external death stimulation signals, resulting in its trimerization, activation of downstream caspase-8 and apoptosis

The extrinsic death receptor pathway, a mechanism initiated by extracellular stimuli to induce apoptosis, contingent upon the interaction between death receptors and their respective ligands. Death receptors (DRs) are transmembrane proteins within the tumor necrosis factor receptor (TNFR) superfamily and include a specific region known as the death domain (DD). Upon stimulation, death ligands on the cell surface promote the trimerization of death receptors, leading to the aggregation of DDs and the initiation of apoptosis (Han et al. 2023). FasL induces Fas trimerisation and binds to it, leading to DD aggregation of the three Fas to attract an additional protein FADD with the same DD. FADD collects pro-caspase-8 through the death effector domain DED to form the death-inducing signalling conduction complex (DISC), which then cleaves pro-caspase-8 and initiates the subsequent caspase cascade to cause apoptosis (Kischkel et al. 1995; Ranjan and Pathak 2024). Furthermore, similar to the aforementioned interactions, TNF ligands engage with the TNFR1 receptor, recruiting TRADD, which subsequently recruits and activates tumor necrosis factor receptor-associated factor 2 (TRAF2), receptor-interacting protein kinase (RIPK), and cIAP1 to form Complex I. On one hand, Complex I can inhibit apoptosis by activating the nuclear factor-kappa B (NF-κB) pathway, thereby suppressing the activation of caspase-8. On the other hand, Complex I can give rise to two variants, Complex IIA (comprising TRADD, FADD, and caspase-8) and Complex IIB (consisting of RIPK1, FADD, and caspase-8), both of which ultimately trigger the activation of caspase-8 leading to apoptosis (Mahmood and Shukla 2010; Pan et al. 2021) (Fig. 1).

### Apoptosis and DKD

#### Podocyte

Modern studies have found that in patients with DKD, the onset of podocyte apoptosis tends to coincide with the occurrence of hyperglycaemia, and that any reduction in podocyte density precedes the onset of proteinuria, which suggests that glycolipid toxicity may be the basis of stimulating apoptosis signal transduction and subsequent podocyte damage (Bhatti and Usman 2015; Erekat 2022). In the HG environment, a multitude of cellular responses, including glucose and lipid metabolism disorders and oxidative stress, stimulate the onset of ERS, thereby leading to podocyte apoptosis. Shen et al*.* found that overexpression of long-stranded non-coding RNA (TUG1) mediated ERS in podocytes and further led to increased expression of CHOP and expanded podocyte apoptosis (Shen et al. 2019). In the study conducted by Zhang et al., HG treatment in podocytes led to the upregulation of CHOP, GRP78, and caspase-12 proteins, along with a significant increase in the rate of podocyte apoptosis. This indicates that HG mediates podocyte apoptosis through the ERS pathway. The specific mechanism may involve the activation of Cyclin-dependent kinase 5 (Cdk5) under conditions of HG-induced ERS. This activation induces the phosphorylation of MEKK1 at the Ser280 site in podocytes, which in turn activates downstream JNK phosphorylation, thereby promoting the occurrence of apoptosis (Zhang et al. 2017). Fan and colleagues have reported an increase in ERS-related markers (GRP78, CHOP, and PERK), as well as the ER-resident protein reticulon (RTN) 1A in podocytes of the kidneys from DKD mice. The specific mechanism suggests that under HG conditions, there is an overexpression of RTN1A in podocytes, which induces ERS. Interestingly, CHOP has a positive feedback effect on RTN1A, exacerbating ERS and promoting the expression of Bax and caspase-3, leading to the induction of apoptosis. Therefore, in the injury of podocytes in DKD, ERS and its regulatory factor RTN1A may play a significant role (Fan et al. 2017).

Podocytes require high energy demands to maintain the organisation and motility of cytoskeletal and extracellular matrix proteins; therefore, podocytes require a large number of mitochondria for energy supply and are also more susceptible to the mitochondrial apoptosis pathway. The glycolipid toxicity caused by HG state will lead to mitochondrial dysfunction, affect mitochondrial dynamics and biogenesis, and finally lead to mitochondrial damage, which results in the release of Cyt *C* to participate in apoptosis (Hu et al. 2020). Ma et al.'s experiments revealed that HG induces significant mitochondrial fragmentation in podocytes, accompanied by increased levels of Cyt *C* and caspase-3, leading to podocyte apoptosis. Further investigation in DKD rats demonstrated both podocyte injury and elevated expression of RING-finger protein 166 (RNF166). The knockout of RNF166 resulted in the suppression of Cyt *C*, caspase-3, and caspase-9 expression, a decrease in the expression of the mitochondrial fission molecule dynein-related protein 1 (Drp1), and an improvement in the expression of mitochondrial fusion proteins mitofusin-1 (Mfn1) and Mfn2. These changes led to a reduction in renal dysfunction and podocyte injury. Moreover, high expression of RNF166 was observed in renal biopsies from DKD patients, with particularly prominent levels in podocytes. Therefore, the experiments confirm that the upregulation of RNF166 under HG conditions is prone to disrupt mitochondrial dynamics, promote apoptosis of podocytes mediated by the mitochondrial pathway, and accelerate the progression of DKD (Hongbo et al. 2021). In addition, damaged mitochondria produce large amounts of reactive oxygen species (ROS), which on the one hand can collect Bax to reduce mitochondrial membrane permeability, exacerbate mitochondrial damage and Cyt C release and thus aggravate apoptosis (Yang et al. 2017). On the other hand, ROS activate the JNK/p38 mitogen-activated protein kinase (MAPK) pathway, which leads to the activation of caspase-3 to promote apoptosis (Sánchez-de-Diego et al. 2019; Yue and López 2020). Thioredoxin interacting protein (TXNIP) plays an important role in ROS regulation, and Shah et al. demonstrated that TXNIP deficiency leads to a reduction in ROS and inhibition of podocyte apoptosis (Shah et al. 2015).

#### Renal tubular cell

In addition to podocytes, an increasing number of experiments in recent years have identified apoptotic cells in the tubular epithelium of diabetic kidneys, confirming the important role of apoptosis of tubular cells, especially RTECs, in DKD (Brezniceanu et al. 2008; Kumar et al. 2004). There are albuminuric and non-albuminuric nature of DKD, whereas the latter is characterised by tubulointerstitial damage and fibrosis without significant glomerular lesions. In addition, when DKD further deteriorated into ESRD, a significant proportion of patients do not have significant proteinuria but mainly show tubular damage (Mottl et al. 2013). Apoptosis of RTECs exacerbates tubular atrophy and tubulointerstitial fibrosis, and thus it plays an important role in the development of DKD.

Activation of the mitochondrial and endoplasmic reticulum pathways in the HG environment represents a significant modality for the induction of apoptosis in RTECs. It is worthy of note that RTN1A, which affects ERS and is involved in podocyte apoptosis, was described above. Furthermore, Xie et al. have suggested that RTN1A, which affects ERS, is also involved in HG-induced apoptosis in RTECs. In DKD rats and HG induced RTECs, HG promotes RTN1A overexpression, which in turn exacerbates ERS and induces tubular injury, apoptosis, and interstitial fibrosis. It is noteworthy that studies on the mechanism by which RTN1A overexpression induces DKD progression have revealed that RTN1A is involved in the protein complex that makes up ER-mitochondria contacts (EMC). Overexpression of RTN1A alters the EMC in RTECs by affecting several outer mitochondrial membrane proteins. This leads to enhanced endoplasmic reticulum-mitochondria crosstalk, which in turn induces mitochondrial dysfunction during the escalation of ERS. Consequently, this results in a reduction of mitochondrial DNA (mtDNA), an increase in mitochondrial fragmentation, and the upregulation of Cyt C expression. In light of these findings, the present study broadens the scope of RTN1A research, offering a novel perspective on apoptosis inhibition in DKD treatment through the lens of EMC in RTECs and ensuing ER-mitochondrial crosstalk (Xie et al. 2022). Huang et al. have reported that HRD1, as an E3 ubiquitin ligase, facilitates the ubiquitination and degradation of eIF2α, thereby inhibiting the PERK/eIF2α/ATF4/CHOP pathway activated by ERS and alleviating apoptosis. In the kidneys of DKD rats and in HG-cultured renal tubular epithelial cells (HKC-8), low expression of HRD1 was observed, along with high expression of eIF2α and apoptosis-related markers (caspase-3, Bax). Furthermore, Transfection of Myc-eIF2 into HKC-8 cells attenuates the cytoprotective effect of HRD1. In summary, the ubiquitination of eIF2α mediated by HRD1 disrupts the downstream pathways of ERS, safeguarding RTECs from HG-induced apoptosis (Huang et al. 2017c).

Verzola et al*.* demonstrated that a HG concentration of 30 mmol/L induced ROS production and inhibited NF-κB activity, thereby inhibiting anti-apoptotic XIAP protein activity downstream of NF-κB, resulting in increased apoptosis in human proximal renal tubular cells (HK-2 cells) (Verzola et al. 2004). Chen et al*.* found that decreasing the expression of ROS and NADPH oxidase 4 (Nox4) in HG-induced HK-2 cells significantly inhibited apoptosis (Chen et al. 2021). Allen et al*.* demonstrated that increased levels of ROS in HG-induced RTECs led to an increase in peroxynitrite and activation of caspase-3 for apoptosis. Significant inhibition of apoptosis was seen when caspase-3 inhibitors were added, thus demonstrating that caspase-3 activation is a major mediator of HG-induced apoptosis in RTECs (Allen et al. 2003). In addition, the clinical study of Hong et al*.* found that the specificity of mitochondrial fragmentation existed in renal tubular cells of DKD patients, but not in podocytes. The accumulation of damaged mitochondria led to loss of MMP and increase in ROS in patients, thereby inducing apoptosis in renal tubular cells (Jiang et al. 2019).

Despite the abundance of research examining the correlation between apoptosis and DKD, several key questions remain unanswered. Mfn1 and Mfn2-mediated mitochondrial fusion and Drp1-mediated mitochondrial fission play pivotal roles in maintaining MMP, safeguarding mitochondrial homeostasis, and preventing apoptosis. However, further investigation is needed to elucidate their expression patterns in kidney-associated cells under HG conditions. Furthermore, dynamic organelle crosstalk through direct interactions at membrane contact sites has become an important regulator of cellular homeostasis. The molecular regulation of EMC in the HG state, the frequency and distance of EMC in normal and DKD kidney-associated cells, as well as the effects of ER-mitochondrial crosstalk in association with EMC on the kidneys of patients with DKD deserve to be explored in depth.

## Autophagy

### Overview of autophagy

Autophagy, denoted as type II PCD, entails the degradation of misfolded proteins and damaged organelles into basic molecular constituents by lysosomes, serving as raw materials and energy sources for cellular metabolism (Yamamoto and Matsui 2024). Upon cellular stimuli, the UNC-51-like kinase 1 (ULK1) complex activates through multiple pathways, subsequently triggering the phosphatidylinositol-3-kinase (PI3K) complex, including Beclin1. This complex, located at phagosomal nucleation sites, orchestrates phagosome nucleation from the ER or adjacent organelles like the Golgi apparatus. Nucleated phagophores expand and engulf cytoplasmic contents to form autophagosomes, regulated by the autophagy-related gene (Atg)12 conjugation system and microtubule light chain protein (LC3)-Phosphatidylethanolamine (PE) conjugation system. LC3 protein cleavage by Atg4 generates LC3-I, which subsequently associates with PE to form LC3-II, marking autophagy activation and progression. Microtubules (MTs) facilitate autophagosome trafficking to lysosomes, culminating in fusion and substrate degradation (Liu et al. 2024a).

Autophagy modulation primarily hinges on nutrient-sensing pathways, involving mammalian target of rapamycin (mTOR), Adenosine 5'-monophosphate-activated protein kinase (AMPK), and Silent Information Regulator 1 (SIRT1). Under conditions of starvation or nutrient deficiency, mTOR is inactivated, and AMPK is activated, thereby promoting autophagy. Conversely, when nutrients are plentiful, this process is reversed to inhibit autophagy. Additionally, AMPK can suppress the activity of the mTOR activator Rheb by activating Tuberous Sclerosis 1/2 (TSC1/2) (Parmar et al. 2022; Yamamoto and Matsui 2024). SIRT1 activates autophagy by deacetylating Forkhead box O transcription factors (FOXOs) under starvation conditions. Furthermore, SIRT1 forms a molecular complex with Atg5, 7, and 8, bolstering autophagy (Lee 2019).

### Autophagy and apoptosis

In the regulation of cell death, autophagy and apoptosis exhibit three interrelations: cooperation, promotion, and antagonism. In terms of cooperation, severe autophagy induces autophagy-mediated cell death. Additionally, pathways related to autophagy and apoptosis can complement each other or serve as alternative pathways to induce cell death (Ando et al. 2021; Mariño et al. 2014). Interestingly, under HG stimulation, the activation of apoptotic signals can also induce the autophagy pathway, leading to cell death. However, in most cases, apoptosis inhibits autophagy-mediated cell death (Ouyang et al. 2014). The promotive effect refers to the capacity of autophagy to sustain intracellular ATP levels during nutrient deprivation, thereby meeting the requirements for the activation of apoptotic signals (Ito et al. 2005). The antagonistic relationship refers to mild autophagy confers cell protection by scavenging ROS, preserving mitochondrial homeostasis, and alleviating ERS, inflammation, and oxidative stress (D'Arcy 2019).

The mutual regulation of autophagy and apoptosis chiefly manifests in the Bcl-2/Beclin1 complex formation. Beclin1, with a BH3 motif, recruits free Bcl-2 for complex assembly, inhibiting autophagy, albeit Bcl-2 retains anti-apoptotic function at this juncture. Competitive binding of Bax and Atg12 to Bcl-2 liberates Beclin1, activating autophagy while potentiating apoptosis. Furthermore, caspase-3 inactivates Beclin1, curbing autophagy, whereas Beclin1 heightens apoptosis by upregulating caspase-9 activity (D'Arcy 2019; Mariño et al. 2014).

**Fig. 2:**
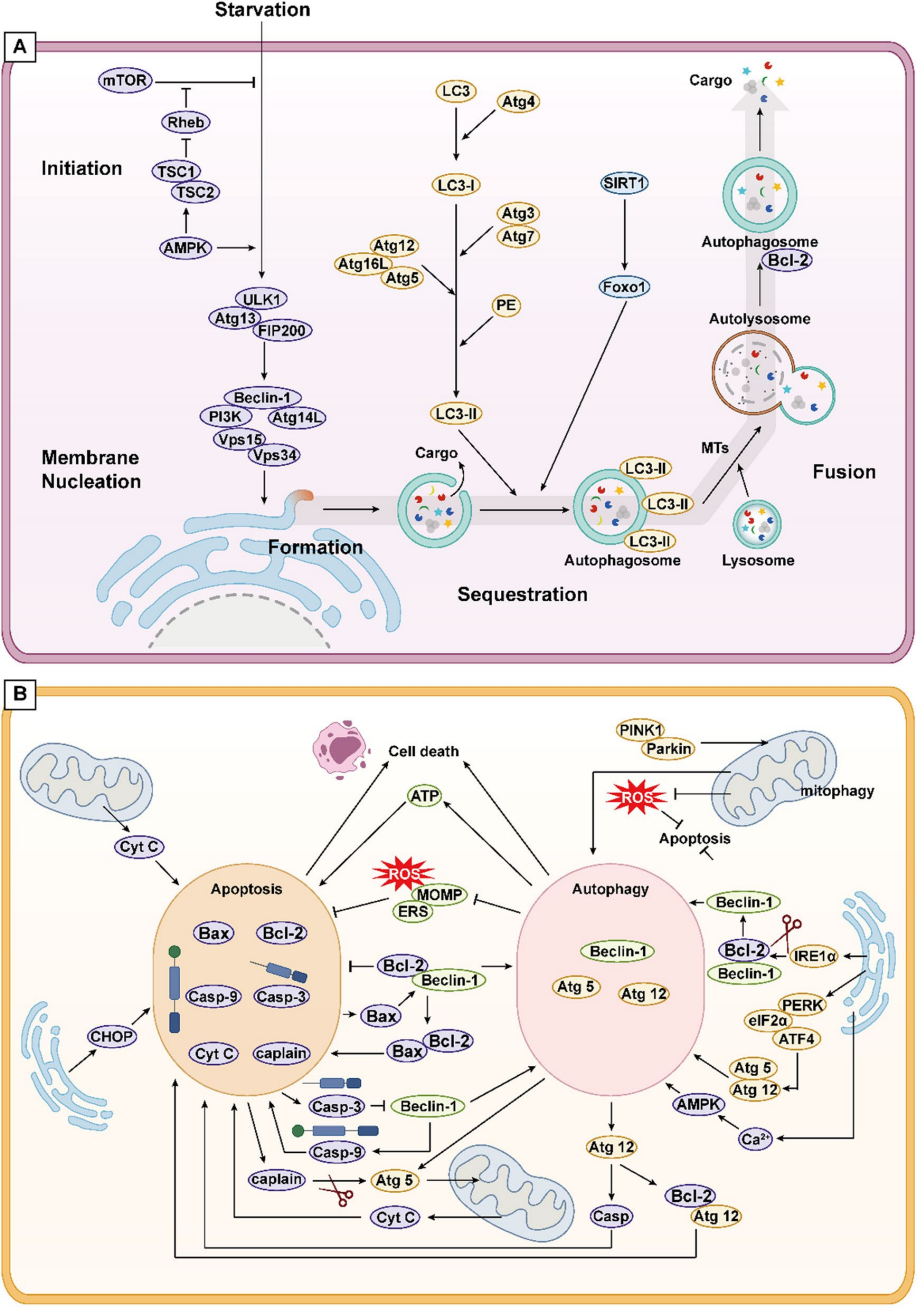
**A** The autophagy process includes the following steps: initiation, formation, fusion and cleavage. External stimuli, such as starvation, induce the formation of ULK1 complex by affecting AMPK and mTOR pathways, mediate the activation of PI3K complex on the membrane of organelles such as endoplasmic reticulum, and induce the formation of phagophore. Subsequently, the phagophore passes through two ubiquitin-like Atg-coupled systems to form mature autophagosome, which fuse with lysosomes through the transport of MTs to form autolysosome, so as to degrade cargoes and complete the autophagy process. **B** There are three relationships between autophagy and apoptosis, namely, cooperation, promotion and confrontation. The Bcl-2/Beclin1 complex, caspase protein family and Atg protein family are involved in the crosstalk between the two. In addition, mitochondria and endoplasmic reticulum can cause apoptosis or selective autophagy, thereby regulating the crosstalk between them

Both apoptosis and autophagy play important roles in maintaining mitochondrial and ER homeostasis. Mitophagy refers to the aggregation of PTEN induced kinase 1 (PINK1) at the outer mitochondrial membrane and activation of the ubiquitin ligase Parkin when the mitochondria are damaged, which is subsequently recognised by p62 (autophagy linker proteins) and degraded after transport to the autophagosome via LC3 (Saito and Sadoshima 2015). ERS augments autophagy by activating Atg5, 12, primarily via the PERK/elF2α/ATF4 pathway. IRE1α and Ca^2+^ activation disrupts the Bcl-2/Beclin1 complex, instigating autophagy (Senft and Ronai 2015). Furthermore, the accumulation of ROS triggers autophagy, and mitophagy in turn reduces ROS levels, thereby diminishing the incidence of apoptosis (Hinchy et al. 2018; Li et al. 2015) (Fig. 2).

### Autophagy and DKD

Autophagy is a protective mechanism regulated by nutritional and stress signals in organisms. In the early stages of DKD, autophagy helps suppress excessive oxidative stress and inflammation by degrading unfolded proteins and damaged organelles. However, as DKD progresses, the nutritional imbalances and various stress responses induced by a hyperglycemic environment exceed the regulatory capacity of autophagy. This leads to autophagic dysfunction, which in turn causes damage to renal cells. This cellular damage further triggers stress responses, creating a positive feedback loop that exacerbates the progression of DKD.

#### Podocyte

Podocytes, highly specialized cells, maintain elevated autophagic activity in vivo to uphold cellular homeostasis. Prolonged HG stimulation-induced autophagy imbalance readily induces podocyte damage. Impaired autophagic flux and decreased LC3-II and Beclin1 expression in DKD rat podocytes were reported by Zhang et al. (2023c). Hartleben et al. illustrated ERS emergence and proteinuria in Atg5-deficient podocyte-bred rats, evidencing podocyte loss and delayed glomerulosclerosis, highlighting autophagy's pivotal role in podocyte homeostasis regulation (Hartleben et al. 2010). Similarly, Tagawa et al. observed the presence of autophagy deficiency and podocyte damage in both patients and rats with diabetes mellitus with massive proteinuria. However, no autophagy deficiency was noted in podocytes of patients and rats with minimal or no proteinuria. To further investigate, they used rats with Atg5-specific deficiencies in podocytes, induced by a high-fat diet (HFD) as a model of diabetes. These rats also exhibited podocyte loss and significant proteinuria. This suggests that while hyperglycemia induces glomerular injury with minimal proteinuria initially, the progression of DKD leads to deficiencies in podocyte autophagy, resulting in substantial podocyte loss and consequently, significant proteinuria. Interestingly, a large number of damaged lysosomes were observed in the podocytes of DKD rats with massive proteinuria and in HFD-fed rats with Atg5-specific podocyte deficiency. This lysosomal damage further disrupted autophagy processes. Tagawa et al.'s findings indicate that maintaining autophagic homeostasis is crucial for preserving normal podocyte function, thereby slowing disease progression and reducing proteinuria in DKD (Tagawa et al. 2016).

What are the mechanisms underlying impaired podocyte autophagy in an HG environment? The imbalance in podocyte autophagy may be related to the alterations in the nutrient-sensing pathways mentioned above. Liu et al. demonstrated Placenta-derived Mesenchymal Stem Cells' (P-MSCs) SIRT1 and FOX01 upregulation, enhancing podocyte LC3 and Beclin1 expression to reinforce autophagy, thereby reducing glomerular matrix deposition (Liu et al. 2024b). Xu et al. demonstrated that metformin activates the SIRT1/FOXO1 pathway, promoting the expression of LC3-II and Beclin1, which reduces glomerular basement membrane thickness and foot process fusion in DKD rats. Furthermore, SIRT1 inhibitors were found to disrupt the renal protective effects of metformin by reducing autophagy (Xu et al. 2020a). Similarly, Ren et al. found that metformin’s activation of the AMPK/SIRT1-FoxO1 pathway promotes autophagy, reduces oxidative stress, and enhances cell viability in high-glucose-induced rat mesangial cells (Ren et al. 2020). Lai et al. demonstrated that administering irisin to podocytes in DKD mice inhibited the PI3K/AKT/mTOR signaling pathway, promoting the restoration of autophagosome numbers and podocin expression. This intervention alleviated severe proteinuria and mitigated glomerular pathological damage in the mice. The study also suggested that decreased plasma irisin levels are associated with deteriorating renal function in DKD patients. Plasma irisin levels were significantly lower in DKD patients compared to those without DKD, and levels were lower in the massive proteinuria group compared to the microproteinuria group. This supports the correlation between podocyte autophagy levels and proteinuria as described above (Lai et al. 2023).

Beyond nutrient-sensing pathways, apoptosis also has a crosstalk relationship with autophagy. Wogonin targeted Bcl-2 to heighten HG-induced autophagy in podocyte line MPC5 cells, as evidenced by increased LC3-II, Beclin1, and Atg7 expression, and diminished podocyte-specific marker WT-1, slit diaphragm protein (SD), and caspase-3 expression (Liu et al. 2022b).

#### Renal tubular cell

Unlike podocytes, RTECs exhibit low levels of basal autophagy under normal conditions. RTEC active transport depends heavily on abundant energy, necessitating autophagic homeostasis for survival in nutrient-deficient settings. Atg7-specific RTEC knockout in DKD mice correlated with heightened renal tubular injury, fibrosis, and albuminuria, alongside negative ULK1/LC3 and renal fibrosis correlation, affirming autophagy's protective role in DKD (Ma et al. 2020). Similar to podocytes, the imbalance of autophagy in RTECs is also related to alterations in nutrient-sensing pathways. Meng et al. showcased decreased autophagic activity in diabetic mice and HG-induced human renal proximal RTECs, with Klotho inducing autophagic activity via AMPK activation to safeguard RTECs (Xue et al. 2021). Li et al. found impaired autophagy in the kidneys of DKD mice and in high-glucose-induced HK-2 cells. They discovered that vitamin D increases Ca^2+^ concentration in a vitamin D receptor-dependent manner, promoting the activation of calcium-calmodulin dependent protein kinase kinase 2 (CAMMKK2). CAMMKK2, acting as an upstream kinase of AMPK, phosphorylates the AMPK/ULK1 pathway, restoring autophagy activity and potentially reducing inflammation, thereby delaying DKD progression and HK-2 cell injury (Li et al. 2022a). Kitada et al. observed that a very low protein diet inhibited mTOR activity in RTECs of diabetic Wistar fatty rats, increased LC3-II expression, restored autophagy, reduced tubulointerstitial injury, and slowed the progression of advanced DKD (Kitada et al. 2016).

Additionally, selective autophagy in RTECs is also involved in the treatment of DKD. As RTECs are rich in mitochondria, maintaining mitophagy homeostasis is essential for preserving their normal structure and function. Wang et al. proposed that ectopic ceramide (CER) synthesized by ceramide synthase 6 (Cers6) in HG-induced RTECs can interact with PINK1, inhibiting the PINK1/Parkin pathway. This inhibition leads to mitochondrial homeostasis disruption, contributing to proteinuria and interstitial fibrosis in DKD patients (Wang et al. 2023a). Liu et al. observed a high expression of TNFAIP8L1/TIPE1 in RTECs from DKD patients and mice. They found that specific knockdown of TIPE1 in mouse RTECs rescued cell injury, alleviated EMT, and reduced renal fibrosis. Further studies in selected DKD mice and HK-2 cells revealed that TIPE1 interacts with the mitophagy receptor prohibitin 2 (PHB2), promoting its degradation via the ubiquitin-protein proteasome pathway. This process subsequently downregulates Pink1, Parkin, Atg12, and LC3-II expression, upregulates MMP, inhibits mitochondrial autophagy, and promotes apoptosis, thereby accelerating DKD progression. Therefore, TIPE1 may act as a potential inhibitor of mitochondrial autophagy in RTECs (Liu et al. 2022a). The mitochondria-associated endoplasmic reticulum membrane (MAM) is a crucial platform that regulates mitophagy, mitochondrial dynamics, and Ca^2+^ signaling to maintain cellular homeostasis. Phosphofurin acidic cluster sorting protein 2 (PACS-2) plays a role in regulating MAM formation. Li et al. found that PACS-2 was significantly reduced in the renal tubules of patients with DKD, with its expression negatively correlated with the severity of tubulointerstitial lesions and positively correlated with renal function. This correlation may be due to PACS-2 binding to Beclin-1, facilitating its localization to the MAM and promoting Pink1-mediated mitophagy restoration, thereby maintaining mitochondrial homeostasis (Li et al. 2022b). Yang et al. observed severe ER autophagy impairment in STZ-induced diabetic mouse RTECs, with notable membrane transport protein for autophagy, PACS-2, and ER autophagy receptor, FAM134B, downregulation, affirming ER autophagy's RTEC-protective role (Yang et al. 2023b). In addition, Han et al. reported that lipophagy, a form of autophagy targeting lipid droplets, was reduced in renal tubular cells from DKD patients and mice, as well as in HG-induced HK-2 cells. When autophagy-promoting lipocalin receptor activators were used, a significant reduction in renal lipotoxicity and ectopic lipid deposition was observed, demonstrating that lipophagy plays a protective role in mitigating renal injury in DKD (Han et al. 2021).

However, the understanding of autophagy's role in DKD remains limited. Autophagy is recognized as having a double-edged sword effect in DKD development: while moderate autophagy can inhibit renal injury, both insufficient and excessive autophagy can exacerbate cellular dysfunction. Currently, there is a lack of research on how to precisely regulate the extent of autophagy to maintain it within an "optimal range." It has been suggested that this "optimal zone" might be related to the stage and severity of diabetes and the specific cell type involved, but there is still a gap in knowledge at the molecular level. Additionally, mitochondrial dysfunction and ers responses play significant roles in the development of DKD. However, comprehensive insights into how HG regulates upstream pathways to induce these cellular stress responses and how these stresses restore autophagic activity via downstream molecules are still lacking. Future studies need to elucidate key molecules and their interactions in autophagy-related pathways, as well as explore novel strategies for DKD intervention through the modulation of autophagy.

## Ferroptosis

### Overview of ferroptosis

Ferroptosis is a recently identified form of PCD that is dependent on iron-mediated oxidative damage. It is fundamentally driven by metabolic disruptions caused by the excessive intracellular accumulation of lipid peroxides (LPO), which are catalyzed by excess iron ions, leading to the production of large amounts of lipid ROS that induce cell death (Tang et al. 2021).

The formation of iron overload is a critical prerequisite for ferroptosis. Fe^3+^ enters the cytoplasm through its binding to transferrin (TF) and transferrin receptor 1 (TFR1), and is subsequently reduced to Fe^2+^ by enzymes such as six-transmembrane epithelial antigen of prostate 3 (STEAP3) (Philpott and Jadhav 2019). Divalent metal transporter 1 (DMT1) translocates Fe^2+^ to the labile iron pool (LIP) in the cytoplasm. Excess Fe^2+^ is either oxidized to Fe^3+^ by ferritin heavy chain (FTH) within ferritin for storage or exported from the cell via ferroportin (FPN). An imbalance in these processes leads to iron overload, triggering Fenton and Haber–Weiss reactions that generate large amounts of lipid ROS (Philpott and Jadhav 2019; Tang et al. 2021).Free polyunsaturated fatty acids (PUFAs) in the cell membrane, such as arachidonic acid (AA) and adrenic acid (AdA), are attacked by these ROS. This attack causes enzymes such as acetyl coenzyme A synthetase long-chain family 4 (ACSL4), lysophosphatidylcholine acyltransferase 3 (LPCAT3), and lipoxygenases (LOXs) to collectively form toxic AA/AdA-OOH-PE compounds that induce ferroptosis (Bouchaoui et al. 2023; Yang et al. 2016). The system Xc- is a transmembrane protein complex composed of SLC7A11 and SLC3A2, which activates the antioxidant glutathione (GSH) to subsequently activate glutathione peroxidase 4 (GPX4). GPX4 reduces AA/AdA-OOH-PE to non-toxic AA/AdA-OOH-PE, thereby inhibiting ferroptosis (Wu et al. 2021). Therefore, the system Xc-/GSH/GPX4 pathway is crucial in the regulation of ferroptosis, alongside other pathways that regulate lipid metabolism and iron homeostasis.

#### Regulation of lipid metabolism

Lipid metabolism regulation in ferroptosis involves Ferroptosis suppressor protein-1 (FSP1) on the cell membrane and dihydroorotate dehydrogenase (DHODH) on the mitochondrial inner membrane. Both of these can reduce coenzyme Q10 (CoQ10) to its antioxidant form, CoQ10H2, which traps lipid peroxyl radicals that mediate lipid peroxidation, thus inhibiting ferroptosis (Lv et al. 2023b; Madak et al. 2019).

#### Regulation of iron metabolism

Heat shock protein β1 (HSPB1) and iron-sulfur cluster biosynthetic enzyme (NFS1) reduce intracellular Fe^2+^ concentration, thus inhibiting Ferroptosis. HSPB1 inhibits Fe^3+^ entry by suppressing transferrin receptor 1 expression, while NFS1 enhances iron-sulfur cluster content, inhibiting Fe^2+^ release from iron storage molecules (Alvarez et al. 2017; Sun et al. 2015). Nuclear factor-erythroid 2-related factor 2 (Nrf2) crucially regulates redox homeostasis, activating GPX4 expression and NADH dehydrogenase quinone 1 (NQO1) and FTH1 transcription, thereby inhibiting ferroptosis (Abdalkader et al. 2018; Sun et al. 2016).

### Relationship between ferroptosis and autophagy

Increasing evidence suggests that ferroptosis is autophagy-dependent, with overactivated selective autophagy (such as ferritinophagy, mitophagy, and lipophagy) or heightened lysosomal activity leading to intracellular Fe^2+^, Fe^3+^, and LPO accumulation, thereby activating Ferroptosis (Zhou et al. 2020). In addition, Cell autophagy disrupts redox homeostasis, promoting ROS-dependent Ferroptosis, which in turn induces cellular autophagy, establishing a positive feedback loop amplifying ferroptosis (Lee et al. 2023).

#### Selective autophagy

Ferritinophagy is the selective degradation process of ferritin, resulting in the release of free Fe^2+^. FTH1 binds to nuclear receptor co-activator 4 (NCOA4), a cargo receptor responsible for ferritin degradation. NCOA4 transports ferritin to phagolysosomes, where autophagic processes release Fe^2+^ (Mancias et al. 2014). When Fe^2+^ concentration becomes excessively high, NCOA4 is degraded, preventing further ferritinophagy (Jin et al. 2023; Mancias et al. 2015). Thus, an imbalance in ferritinophagy can lead to increased Fe^2+^ levels, which subsequently induce ferroptosis. In addition, imbalances in mitophagy and lipophagy affect the homeostasis of iron ions and lipids, respectively, and promote ferroptosis.

A part of free Fe2 + will enter mitochondria and participate in the electron transfer of enzymatic redox reaction as a necessary auxiliary factor. In the early stage of iron overload, the occurrence of mitophagy isolates Fe^2+^ in autophagy. With the aggravation of iron overload, the mitochondrial damage is aggravated, and a large amount of Fe^2+^ is released into the cytoplasm after the imbalance of mitophagy, which exacerbates the occurrence of ferroptosis (Lee et al. 2023).

Lipophagy, the autophagic degradation of intracellular lipid droplets (LDs) into free fatty acids, which will enter mitochondria to participate in ATP production (Liu and Czaja 2013). Thus when lipophagy is abnormal, it impacts mitochondrial biogenesis and intracellular LPO accumulation, potentially triggering Ferroptosis.

#### Partial regulatory proteins

**Fig. 3 A:**
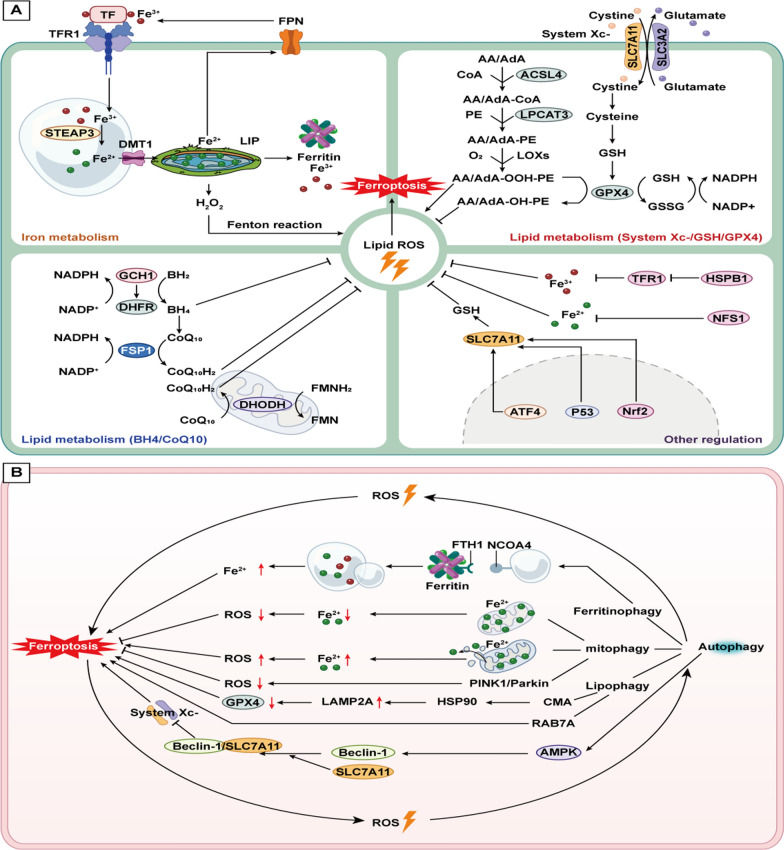
Iron metabolism, lipid metabolism, and other regulatory mechanisms that influence iron-induced cell death. In iron metabolism, Fe^3+^ is transported into the cell via the TF/TFR1 complex and subsequently converted to Fe^2+^. Within the labile iron pool, a portion of Fe^3+^ is exported to the extracellular space, some is stored intracellularly, and the remainder participates in the Fenton reaction with H2O2, leading to the generation of lipid ROS. In lipid metabolism, various axes such as System Xc-/GSH/GPX4, GCH1/DHFR/BH4, FSP1/NADPH/CoQ10, and DHODH/CoQ10 play roles in inhibiting lipid ROS production. It is noteworthy that FSP1, DHODH and GPX4 each exert anti-ferroptosis effects in an independent manner. While GPX4 exists in both mitochondria and the cytoplasm, DHODH can compensate for GPX4 in mitochondria to inhibit mitochondrial lipid peroxidation. However, FSP1 can't compensate with GPX4 in cytoplasm. GCH1, the rate-limiting enzyme for tetrahydrobiopterin (BH4) synthesis, promotes lipophilic antioxidant production. BH4 also stimulates CoQ10 synthesis to resist lipid peroxidation. Other regulatory factors, mainly involving proteins in the nucleus and cytoplasm, exert diverse effects on lipid ROS generation. **B** Crosstalk between ferroptosis and autophagy. Both of them can affect the production of ROS so as to promote each other. Autophagy can affect the occurrence of ferroptosis through ferritinophagy, mitophagy and lipophagy. In addition, ferroptosis can also be suppressed by AMPK influencing system Xc-

Partial proteins also participate in the regulatory relationship between autophagy and ferroptosis. Increased lysosomal membrane protein levels and CMA activity upon erastin treatment inhibit GPX4 degradation, promoting ferroptosis (Wu et al. 2019). Additionally, GSX depletion enhances Ferroptosis and autophagy (Sun et al. 2018). Phosphorylation of Beclin1 by AMPK binds to SLC7A11, inhibiting Xc- system activity and promoting ferroptosis (Song et al. 2018b). ELAV-like RNA-binding protein 1 (ELAVL1) binds to Beclin1, activating ferritinophagy and promoting ferroptosis (Zhang et al. 2018) (Fig. 3).

### Ferroptosis and DKD

In DKD, hyperglycemia induces disorders in glycolipid metabolism, promoting excessive production of intracellular ROS and inhibiting GPX4 activity. This inhibition leads to the overaccumulation of LPO. Additionally, increased oxidative stress, inflammation, and organelle damage, particularly to mitochondria, in the hyperglycemic environment disrupt the regulatory balance of intracellular iron, creating conditions conducive to ferroptosis.

#### Podocyte

HG treatment decreases peroxiredoxin 6 (Prdx6) expression in MPC5 cells, downregulating GSH, GPX4, and SLC7A11 activity while increasing iron accumulation and ROS levels, leading to decreased podocyte viability. Prdx6 overexpression reverses these effects, whereas the ferroptosis inducer erastin addition diminishes Prdx6's protective effects. Thus, targeting the upregulation of Prdx6 to inhibit hyperglycemia-induced ferroptosis in MPC5 cells may offer new therapeutic approaches for DKD (Zhang et al. 2021). Furthermore, Du et al. conducted new investigations into the activation of GPX4 and validated their findings through in vivo experiments using a DKD mouse model. Their research found decreased expression levels of GPX4, SLC7A11, HO-1, SIRT6, and Nrf2, alongside increased ACSL4 expression in the kidneys and HG-cultured podocytes of DKD mice. SIRT6 enhances cellular antioxidant capacity, and its overexpression activates the Nrf2/GPX4 pathway, reverses the expression levels of these proteins, and reduces oxidative stress and ferroptosis. Notably, SIRT6 overexpression increased MMP and improved mitochondrial dysfunction, further inhibiting ferroptosis. The addition of Ferrostatin-1 (Fer-1), a ferroptosis inhibitor, to the HG group yielded results consistent with SIRT6 overexpression, confirming ferroptosis occurrence in these cells. Thus, SIRT6 may alleviate HG-induced ferroptosis, mitochondrial dysfunction, and podocyte injury by targeting the Nrf2/GPX4 pathway (Du et al. 2024). Wu et al. discovered that high fructose intake is prone to inducing ferroptosis in podocytes, possibly due to the upregulation of mitochondrial single-stranded DNA-binding protein 1 (SSBP1). SSBP1 promotes the phosphorylation of p53, which in turn inhibits the expression of downstream SLC7A11 and reduces GPX4 levels, thereby facilitating ferroptosis in podocytes. The natural antioxidant pterostilbene has been shown to inhibit SSBP1, thus reducing the occurrence of ferroptosis. Therefore, targeting SSBP1 could be a potential therapeutic strategy to alleviate podocyte ferroptosis (Wu et al. 2022).

In addition to exploring the regulation of GPX4, other studies have investigated the relationship between ferroptosis and podocyte damage. Xiong et al. observed the accumulation of ROS, Fe^2+^, and the expression of TFR1 and ACSL4 in HG-induced podocytes, indicating ferroptosis occurrence. Treatment with Rhein inhibited the downstream pathway and reversed the expression of these markers. Moreover, Rhein enhanced superoxide dismutase (SOD) activity, reduced malondialdehyde (MDA) levels, and decreased ferroptosis in MPC5 cells by mitigating oxidative stress and lipid peroxidation. Interestingly, Rhein also inhibited α-smooth muscle actin (α-SMA) expression, preventing EMT, suggesting a potential therapeutic approach for DKD (Xiong et al. 2023).

#### Renal tubular cell

In the diabetic environment, oxidative stress imbalance leads to LPO accumulation, which plays a crucial role in ferroptosis. Nrf2, an essential regulator of oxidative stress, influences ferroptosis not only in podocytes but also in renal tubular cells. In a renal biopsy from DKD patients, Seonghun Kim et al. observed significantly lower mRNA expression levels of SLC7A11 and GPX4 in renal tubules compared to non-diabetic samples. Similar results were observed in studies of kidney tissues from DKD and normal mice. However, the addition of Fer-1 to DKD mice improved renal tubular cell survival, reduced intrarenal interstitial edema, and significantly decreased proteinuria, confirming that renal tubular cell damage under diabetic conditions is associated with ferroptosis. In an in vivo study using mice and immortalized rat proximal renal tubular epithelial cells (NRK-52E), Seonghun Kim et al. found that transforming growth factor-β1 (TGF-β1) increased Nrf2 expression while inhibiting SLC7A11 expression, reducing GPX4 synthesis, and promoting LPO accumulation in a time-dependent manner. This led to mitochondrial morphological disruption and ferroptosis. These findings suggest that inhibiting ferroptosis in renal tubular cells could be a promising approach for treating DKD (Kim et al. 2021).

However, there are also related experiments that have proposed contrary opinions. Li et al. observed that HK-2 cells and DKD mice cultured in HG display characteristic ferroptosis mitochondrial morphological changes, iron overload, ROS production, and LPO accumulation. HK-2 cells with specifically knocked down Nrf2 showed increased susceptibility to ferroptosis in HG culture. Conversely, the upregulation of Nrf2 by fenofibrate inhibited ferroptosis-related changes, and slowed DKD progression. Fenofibrate's effects were comparable to those of Fer-1, leading Li et al. to conclude that Nrf2 upregulation could alleviate ferroptosis and protect renal tubular cells (Li et al. 2021). Liu et al. observed that Fer-1 ameliorated EMT-induced overexpression of α-SMA and Vimentin during HG-induced EMT progression in HK-2 cells and in a DKD mouse model. This suggests that HG can induce ferroptosis, promoting EMT progression. Additionally, both in vivo and in vitro models demonstrated that HG activated ERS, with significantly elevated expression of markers ATF6, CHOP, and GRP78. Further studies revealed that ERS activates the XBP1/Hrd1 pathway, leading to Hrd1-mediated ubiquitination and degradation of Nrf2, thereby reducing Nrf2 expression. Low Nrf2 expression increased the sensitivity of HK-2 cells to ferroptosis, as evidenced by elevated levels of ferric ions and MDA and reduced expression of GSH and SLC7A11. Specific knockdown of the Nrf2 gene in HG-induced HK-2 cells led to ferroptosis and EMT, while Fer-1 mitigated these effects. This experiment confirmed the negative regulatory role of Nrf2 in ferroptosis and highlighted the protective potential of targeting ERS to alleviate EMT in renal tubular cells (Liu et al. 2023b). Lu's research team found that SLC7A11 and GPX4 expression in RTECs of DKD patients was lower than in non-DKD patients, confirming the role of ferroptosis. In DKD mice and HG-induced HK-2 cells, empagliflozin activated AMPK/Nrf2, upregulating GPX4, SLC7A11, and FTH1, thereby inhibiting ferroptosis and protecting HK-2 cells. This study suggests a new approach for treating DKD with empagliflozin (Lu et al. 2023).

In addition to empagliflozin, dapagliflozin has been shown to ameliorate renal tubular injury in DKD mice independently of glycemic control. Huang et al. suggested that in HG conditions, inhibition of FPN1 expression in HK-2 cells reduces iron ion efflux, leading to iron overload. Although the expression of iron import and storage proteins, such as TFR1 and FTH1, was not affected, the resulting imbalance contributed to cellular iron overload in HK-2 cells. Dapagliflozin was found to reduce the ubiquitination of FPN1, thereby stabilizing its expression and inhibiting ferroptosis, which subsequently ameliorated renal tubular injury in the HG environment (Huang et al. 2022a). Regarding the crosstalk between ferroptosis and other forms of PCD, calycosin inhibits ferritinophagy, alleviating ROS, and Ferroptosis in HG-induced HK-2 cells, whereas erastin blocks calycosin's therapeutic effect (Huang et al. 2022b).

Despite the evidence linking ferroptosis—facilitated by increased Fe^2^⁺ and attenuated GPX4—with impaired podocyte and renal tubular cell function, the specific mechanisms remain incompletely understood. For instance, there are still gaps in quantifying and assessing changes in intracellular Fe^2^⁺ concentration, and the roles of iron ion transport-related proteins such as FPN1, TFR1, and FTH1 under HG conditions are not fully explored. Although oxidative stress regulatory proteins like Nrf2 and HO-1 are involved in ferroptosis regulation and are potential therapeutic targets for DKD, their specific mechanisms of action are not fully elucidated. It is hypothesized that Nrf2's varying roles may depend on cell type and different upstream molecules. Additionally, although there have been reports on ERS and ferritinophagy, research gaps remain concerning the interplay between the ER and mitochondria under HG influence. For example, it is unclear whether mitophagy regulates ferritinophagy in DKD patients and whether the downstream PERK/eIF2α/ATF4 pathway of the ER affects ferroptosis. These unresolved issues pose challenges for ongoing research and offer critical insights for clinical treatment.

## Pyroptosis

### Overview of pyroptosis

Pyroptosis, a pro-inflammatory form of PCD, is triggered by bacteria, pathogens, or endotoxins. This process involves cell swelling, membrane foaming, and lysis, primarily dependent on the perforation of the cell membrane by the gasdermin (GSDM) protein family (Kovacs and Miao 2017). Inflammatory caspase activation also contributes to pyroptosis, which can be classified into the canonical pathway (caspase-1 dependent) and the non-canonical pathway (non-caspase-1 dependent) (Yu et al. 2021). The classical pathway is currently the main form of induced cellular pyroptosis.

In the classical pathway, Toll-like receptors (TLRs) on the cell membrane recognize pathogen-associated molecular patterns (PAMPs) or damage-associated molecular patterns (DAMPs) from external stimuli. This recognition activates the NF-κB signaling pathway, leading to the transcription of IL precursors and the inactive NOD-like receptor thermal protein domain associated protein 3 (NLRP3). Additionally, DAMPs and PAMPs are specifically recognized by pattern recognition receptors (PRRs), which then recruit apoptosis-related speck-like protein (ASC) and pro-caspase-1. Together, these components form a multiprotein complex known as the inflammasome, with the NLRP3 inflammasome being the most common(He et al. 2016; Malik and Kanneganti 2017).Caspase-1 cleaves GSDMD, generating active GSDMD-N, which aggregates on the cell membrane to form non-selective pores. This aggregation leads to the release of intracellular K^+^, inflammatory factors and DMAPs, which causes a wide range of inflammatory reactions, and finally pyroptosis occurred.

The non-canonical pathway is induced by lipopolysaccharide (LPS), activating caspase-4/5 in humans and caspase-11 in rodents (Matikainen et al. 2020). Activated caspase-4/5/11 cleaves GSDMD, leading to plasma membrane perforation and pyroptosis. Additionally, GSDMD-N acts on the NLRP3/caspase-1 axis, upregulating IL-1β and IL-18 expression. Interestingly, activation of caspase-11 can cleave the channel protein Panexin-1 and induce ATP release, which further activates purinergic ion channel-type receptor 7 (P2X7) to enlarge the pore and promote K + efflux. K + efflux is one of the agonists of NLRP3, so caspase-11 can also activate the classical pathway of pyroptosis (Lu et al. 2020). Moreover, GSDMB binds to the recruitment domain of caspase-4 to promote caspase-4 activity, thereby cutting GSDMD and causing pyroptosis. However, GSDMB is also a substrate for caspase-4, so a negative feedback mechanism may terminate the promotion of the non-classical pyroptosis pathway by GSDMB (Chen et al. 2019a).

### Pyroptosis and its interplay with apoptosis, autophagy, and ferroptosis

Increasing evidence suggests a close relationship and cross-regulation among apoptosis, pyroptosis, and necroptosis. This section explores the interplay between apoptosis and pyroptosis. Functionally, both apoptosis and pyroptosis are involved in immune responses and resistance to bacterial infections. Morphologically, both processes exhibit chromatin condensation, but apoptosis is characterized by cell shrinkage and intact cell membranes, while pyroptosis involves cell swelling, membrane deformities, and pore formation. Both processes are regulated by caspase family members, with apoptotic caspases, including caspase-2/3/6/7/8/9/10, playing a predominant role in apoptosis, and inflammatory caspases, including caspase-1/4/5/11/12/13/14, being critical for pyroptosis (Bertheloot et al. 2021). Recent studies have demonstrated that under specific conditions, Caspase-3/8 can cleave GSDM proteins, facilitating the transition from apoptosis to pyroptosis (Bhat et al. 2023). Wang et al*.* demonstrated that caspase-3 could cleave GSDME to trigger pyroptosis in response to chemotherapeutic drugs or Tumor necrosis factor-α (TNF-α) (Wang et al. 2017b). Caspase-8 induces the formation of ASC spots, followed by the activation of caspase-1 and IL-1β (Fritsch et al. 2019). Hou et al*.* found that caspase-8 and TNF-α cleaved GSDMC to induce pyroptosis. GSDMC/caspase-8 mediated the atypical pyroptosis pathway in cancer cells, transforming apoptosis into pyroptosis to accelerate tumor necrosis (Hou et al. 2020).

The relationship between the NLRP3 inflammasome and autophagy is closely linked through ROS. NLRP3 exerts a dual role on autophagy, while autophagy can inhibit NLRP3 activation. Specifically, the inflammation triggered by the NLRP3 inflammasome leads to the production of ROS, which, as previously mentioned, can induce autophagy. But NLRP3 can inhibit autophagy through E2/ERβ/AMPK/mTOR (Wei et al. 2019). Additionally, ROS can oxidize mtDNA, which then contributes to the activation of NLRP3. Therefore, mitophagy can regulate ROS and maintain mitochondrial homeostasis to inhibit NLRP3 production (Zhong et al. 2018).

**Fig. 4 A:**
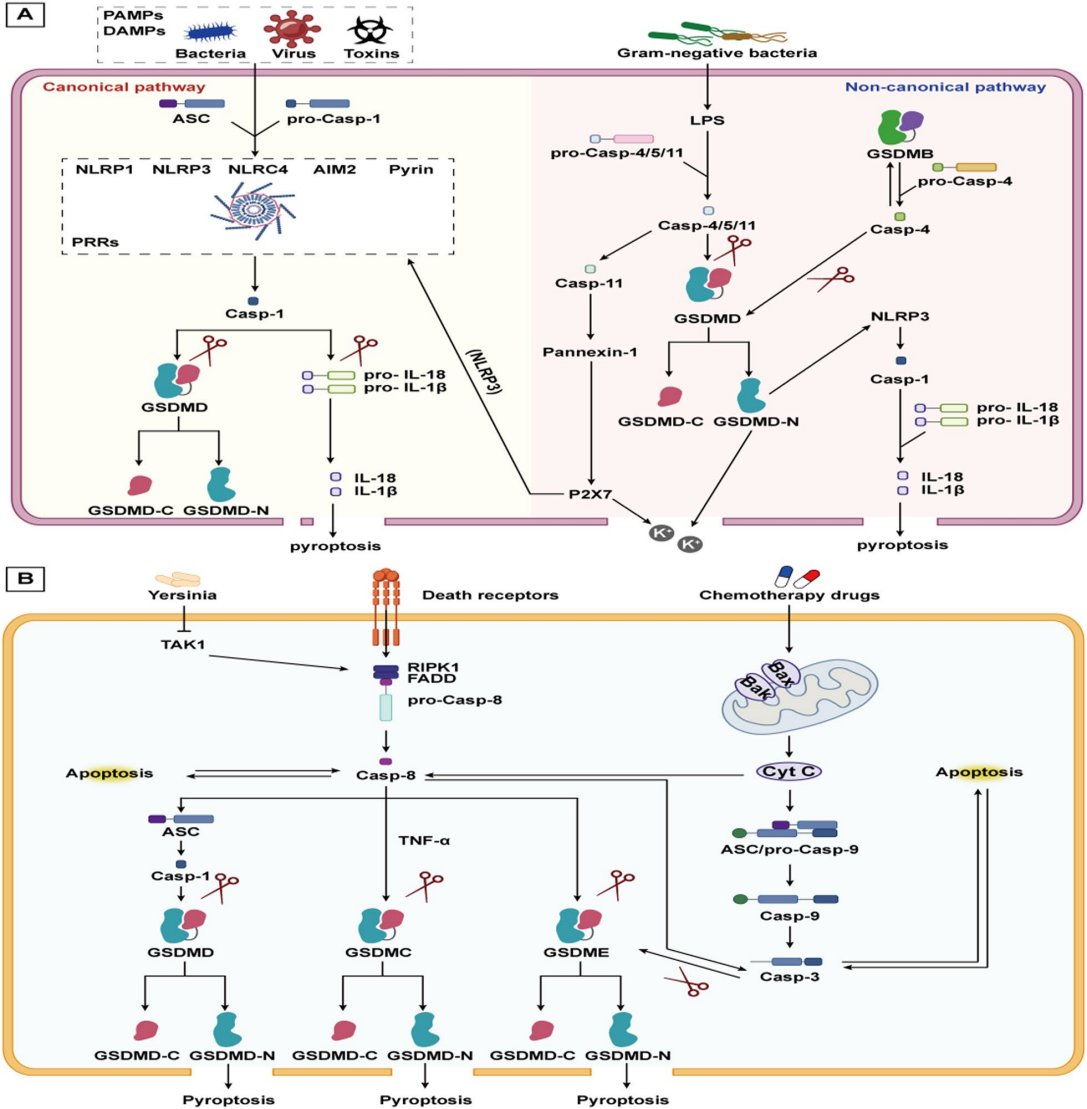
Cellular pyroptosis is divided into two distinct pathways: the canonical pathway and the non-canonical pathway. In the canonical pathway, different inflammasomes, including ASC and pro-caspase-1, are activated under the influence of different external PAMPs and DAMPs. This is followed by the formation of mature caspase-1, which cleaves GSDMD and pro-IL-1β/pro-IL-18, respectively, to form GSDMD-N, which mediates the rupture of the cell membrane and the efflux of IL-1β and IL-18. In the non-canonical pathway, LPS induces pro-caspase-4/5/11 to form mature caspase-4/5/11, which then cleaves GSDMD, thereby inducing focal necrosis. **B** The interaction between pyroptosis and apoptosis. The activation of caspase-8 can be induced by a variety of external stimuli, which in turn can lead to apoptosis. Furthermore, cleavage of GSDMD, GSDMC, and GSDME, which form the N-terminus, can induce pyroptosis. Cyt C, released by mitochondrial damage, activates caspase-3/8, which in turn cleaves GSDME, thereby inducing pyroptosis

Recent studies have highlighted an antagonistic relationship between pyroptosis and ferroptosis. 3-Hydroxy-3-methylglutaryl-coenzyme A reductase (HMGCR), located on mitochondria, promotes GPX4 and CoQ10 expression to inhibit ferroptosis. During pyroptosis, HMGCR shifts to the ER, targeting the NLRP3-caspase-1-GSDMD pathway to induce pyroptosis. When BRCC36 complex increases HMGCR expression, HMGCR binds more tightly to the RPL27 protein on the ER, causing most of the HMGCR to enter the ER and induce pyroptosis, while a smaller portion remains in the mitochondria to inhibit ferroptosis. When BRCC36 is depleted, HMGCR expression decreases, reversing this effect to promote ferroptosis and suppress pyroptosis, highlighting an antagonistic relationship between the two processes (Wang et al. 2024a). It was also evidenced by Hsu et al. that endogenous products of LPO (4-hydroxynonenal) could inhibit NLRP3 inflammasome activation and pyroptosis in macrophages independently of Nrf2 and NF-κB signalling (Hsu et al. 2022) (Fig. 4).

### Pyroptosis in DKD

Pyroptosis, while initially serving as a physiological response promoting immune defense, can exacerbate inflammation and tissue damage when overactivated. However, in the HG environment of DKD, oxidative stress from glycolipid metabolism imbalances activates NLRP3 inflammasome, leading to pyroptosis. This process releases inflammatory cytokines such as IL-18, which exacerbates renal inflammation, cell death, and tissue damage, thereby creating a positive feedback loop that accelerates DKD progression.

#### Podocytes

Pyroptosis in podocytes is often closely associated with HG-induced NLRP3 inflammasome activation and GSDMD-N mediated cell membrane cleavage. Increased expression of caspase-4/11, GSDMD-N, IL-1β, IL-18, and NF-κB, coupled with decreased expression of podocyte markers nephrin and podocin, is observed in HG-cultured podocytes. Knocking down caspase-4 and GSDMD expression significantly suppresses the elevation of these proteins, indicating that under HG conditions, the activation of caspase-4/11 and GSDMD triggers pyroptosis in podocytes. This leads to podocyte loss, thereby exacerbating the progression of DKD (Cheng et al. 2021). Studies have demonstrated elevated caspase-1 and GSDMD expression in renal tissue biopsies from DKD patients. And the addition of Carnosine to HG-induced MPC5 cells and STZ-induced rats significantly inhibited the levels of caspase-1, NLRP3, ASC, and IL-1β, thus increasing cell viability (Zhu et al. 2021).

The occurrence of pyroptosis is closely associated with the activation of the NLRP3 inflammasome. Consequently, various research groups have explored the mechanisms by which inhibition of NLRP3 activation can alleviate podocyte damage. These studies have confirmed that inhibiting the pyroptosis pathway may serve as a potential strategy to expand treatment options for DKD. Sun *et* al.confirmed that the expression of NLRP3, caspase-1 and GSDMD-N in podocytes was up-regulated under HG induction, and the activation of NF-κB was inhibited after the use of breviscapine, followed by inhibition of NLRP3 and pyroptosis (Sun et al. 2023). Similarly, Xu et al. observed an upregulation of pyroptosis markers such as NLRP3, caspase-1, GSDMD, and IL-18 in podocytes damaged by HG induction. Additionally, they found overexpression of tripartite motif-containing 29 (TRIM29) and activation of NF-κB. When TRIM29 expression was silenced, the phosphorylation of NF-κB was inhibited, leading to a reduction in the expression of NF-κB and its downstream target NLRP3, thereby mitigating podocyte injury (Xu et al. 2023). Zhang et al. proposed that dapagliflozin, a commonly used hypoglycemic agent, can exert an anti-pyroptotic effect in the treatment of DKD by targeting pyroptosis. Specifically, it promotes the expression of heme oxygenase-1 (HO-1) in podocytes, which in turn reduces the levels of NLRP3, caspase-1, IL-18, and IL-1β, thereby decreasing cell membrane rupture and mitigating pyroptosis (Zhang et al. 2023d).

#### Renal tubular cells

A large number of studies have found that the expression of both NLRP3 and GSDMD was found to be higher in the renal tissues of DKD patients than that of controls, confirming the occurrence of pyroptosis in the kidneys of DKD patients. Subsequently, HK-2 cells were intervened and cultured in vitro to further explore the mechanism of DKD development under the influence of pyroptosis. Liu et al. cultured HK-2 cells with different glucose concentrations for 24 h and observed that at glucose concentrations of 15 mmol/L and 30 mmol/L, there was a significant increase in the expression of NLRP3, GSDMD-N, IL-1β, IL-18, and caspase-1 compared to the control group. Additionally, HK-2 cells exhibited more pronounced ultrastructural changes characteristic of pyroptosis under these conditions. This experiment explored a new relationship between glucose concentration and HK-2 cell pyroptosis, which is conducive to further research work (Liu et al. 2022c).The experiment of Yuan et al. further suggested that the renal tissues of patients with DKD Yuan et al. further suggested that GSDMD, caspase-1 and IL-1β, which are mainly located in the proximal tubules of the kidney, were elevated in renal tissues of DKD patients. In addition, it was found that GSDMD was positively correlated with 24 h urine protein level and serum creatinine level, and negatively correlated with eGFR, and the above indexes usually reflect the degree of renal tubular damage, so it was proposed that GSDMD expression was positively correlated with renal tubular damage. In HK-2 cells cultured with 30 mmol/L glucose concentration, upregulation of pyrolysis markers including GSDMD was observed. Interestingly, elevated GSDMD suppressed HG-induced expression of Bax and caspase-3 and reduced apoptosis. In a subsequent exploration of upstream targets of GSDMD, knockdown of Toll-like receptor 4 (TLR4) was found to inhibit GSDMD expression and attenuate renal tubular injury improving cell viability. In summary, TLR4 regulation of GSDMD expression initiates the pyrolysis pathway to exacerbate renal tubular injury in DKD patients, so knockdown of TLR4 could be a potential treatment for DKD (Yuan et al. 2022). Xie et al. found that high expression of (Pro)renin receptor (PRR) in renal tubules paralleled the onset of pyroptosis of RTECs, which was positively associated with renal injury in DKD patients. Silencing of PRR inhibits the onset of pyroptosis and overexpression of PRR induces pyroptosis through the JNK pathway (Xie et al. 2024).

Moreover, some studies have demonstrated that in HG-induced HK-2 cells, in addition to the upregulation of pyroptosis-related markers (NLRP3, caspase-1, IL-1β, IL-18), there is often a concurrent upregulation of certain non-coding RNAs, including long noncoding RNAs (LncRNAs) and circular RNAs (circRNAs), as well as a downregulation of their downstream microRNAs (miRs). The correlation between lncRNA/circRNA, miR and NLRP3 was examined by the dual luciferase reporter gene assay, and the results showed that miR could negatively regulate NLRP3 expression in HG-induced HK-2 cells, whereas up-regulated lncRNA/circRNA could down-regulate miR expression, which could then induce the onset of pyroptosis. When specifically knocking down lncRNA/circRNA or overexpressing miR can protect HG-induced HK-2 cells from the damage of pyroptosis. Liu et al. found that upregulated lncRNA metastasis-associated lung adenocarcinoma transcript-1 (MALAT1) negatively regulated miR-30c, which in turn stimulated the expression of NLRP3 and exacerbated the onset of pyroptosis (Liu et al. 2020). Amal Ezzat Abd El-Lateef et al. found that lncRNA nuclear paraspeckle assembly transcript (NEAT2) could promote cellular pyroptosis by inhibiting miR-206 expression (El-Lateef et al. 2022). While Wang et al. found significant up-regulation of Circ_0004951 in renal tissues from DKD patients and HG-intervened HK-2 cells, further studies suggested that Circ_0004951 could down-regulate miR-93-5p expression to activate NLRP3-induced pyroptosis (Wang et al. 2022d). Taken together, LncRNA MALAT1/miR-30c, LncRNA NEAT2/miR-206 and Circ_0004951/miR-93-5p are all expected to be potential targets for clinical targeting of RTEC pyroptosis to treat DKD.

However, there are still limitations in the current studies: elevated blood glucose in the context of DKD activates NLRP3 to induce pyroptosis, and most of the current studies have involved the impression of NLRP3 on its downstream pathways; however, the mechanisms involved in the activation of NLRP3 have not been fully elucidated. Although studies have argued for a possible role of pro-inflammatory factors such as IL-18 in this process, exactly how ASCs are recruited and form the NLRP3-ASC complex, and whether other potential intracellular signals (e.g., changes in membrane markers, ERS, mitochondrial functional status, lysosomal rupture,) are involved in the activation of the NLRP3 inflammasome, all of these need to be further explored. Secondly, the role of other potential inflammasomes (e.g. NLRP1, NLRC4) in podocytes and RTECs under high glucose state and their specific regulatory mechanisms in the process of pyroptosis in the above cells need to be further investigated. Finally, the inter-crosstalk between pyroptosis and apoptosis, autophagy and ferroptosis, as addressed above, needs to be further explored in DKD as well. As observed by Yuan et al., TLR4/GSDMD mediates the potential switching mechanism between pyroptosis and apoptosis, but the specific target of the switch and the related pathways remain unclear.

## Necroptosis

### Overview of necroptosis

Necroptosis refers to an "alternative" form of cell death that occurs when apoptosis is inhibited. While it shares morphological characteristics with necrosis, such as plasma membrane rupture, organelle swelling, and nuclear disintegration, it is regulated by signaling pathways similar to those of apoptosis (Galluzzi et al. 2017).

As detailed in the "Overview of Apoptosis" section, Complex I and Complex IIa/b dictate the fate of cell survival or apoptosis. When caspase-8 is inactivated or suppressed, the apoptotic process is impaired. In this scenario, RIPK1 within the Complex IIb and the abundantly present RIPK3 in the cell undergo mutual phosphorylation, subsequently activating the mixed-lineage kinase domain-like protein (MLKL) to jointly form the necrosome (Yuan et al. 2019). Activated MLKL forms oligomeric complexes that translocate to the plasma membrane, causing changes in membrane permeability, leading to the influx of Mg^2+^ and Ca^2+^ and the efflux of K^+^, accompanied by the release of DAMPs, ultimately resulting in inflammatory necrosis (Weber et al. 2018). Moreover, the necrosome can phosphorylate the mitochondrial protein phosphatase PGAM5, activating Drp1, which leads to mitochondrial lysis and excessive ROS accumulation, further exacerbating cell death (Wang et al. 2012). Additionally, the activated receptors FAS and TRALR can promote the phosphorylation of RIPK1 and RIPK3, thereby mediating the occurrence of necroptosis. In macrophages, double-stranded RNA (dsRNA) and LPS can also induce necroptosis by phosphorylating RIPK3 through a series of reactions (Yang et al. 2023a).

### The interplay among necroptosis, apoptosis, and pyroptosis

Host cells, when stimulated by external viruses or bacteria, induce apoptosis, pyroptosis, and necroptosis to eliminate pathogens. These processes can either promote or complement each other, reflecting a complex interplay in cellular demise. Numerous studies have suggested that caspase-8 acts as a switch between necroptosis, apoptosis, and pyroptosis (Bertheloot et al. 2021; Fritsch et al. 2019). The role of caspase-8 in regulating apoptosis and pyroptosis has been elucidated previously, with active caspase-8 capable of cleaving RIPK1 and RIPK3, thereby preventing cells from undergoing necroptosis (Ashida et al. 2020). It has also been proposed that upon TNF activation, caspase-8 cleaves CYLD to promote cell survival. When a substitution mutation occurs at the CYLD site, caspase-8 loses its ability to cleave CYLD, causing cells to transition from survival to necroptosis (O'Donnell et al. 2011).

### The interplay among necroptosis, autophagy, and ferroptosis

**Fig. 5:**
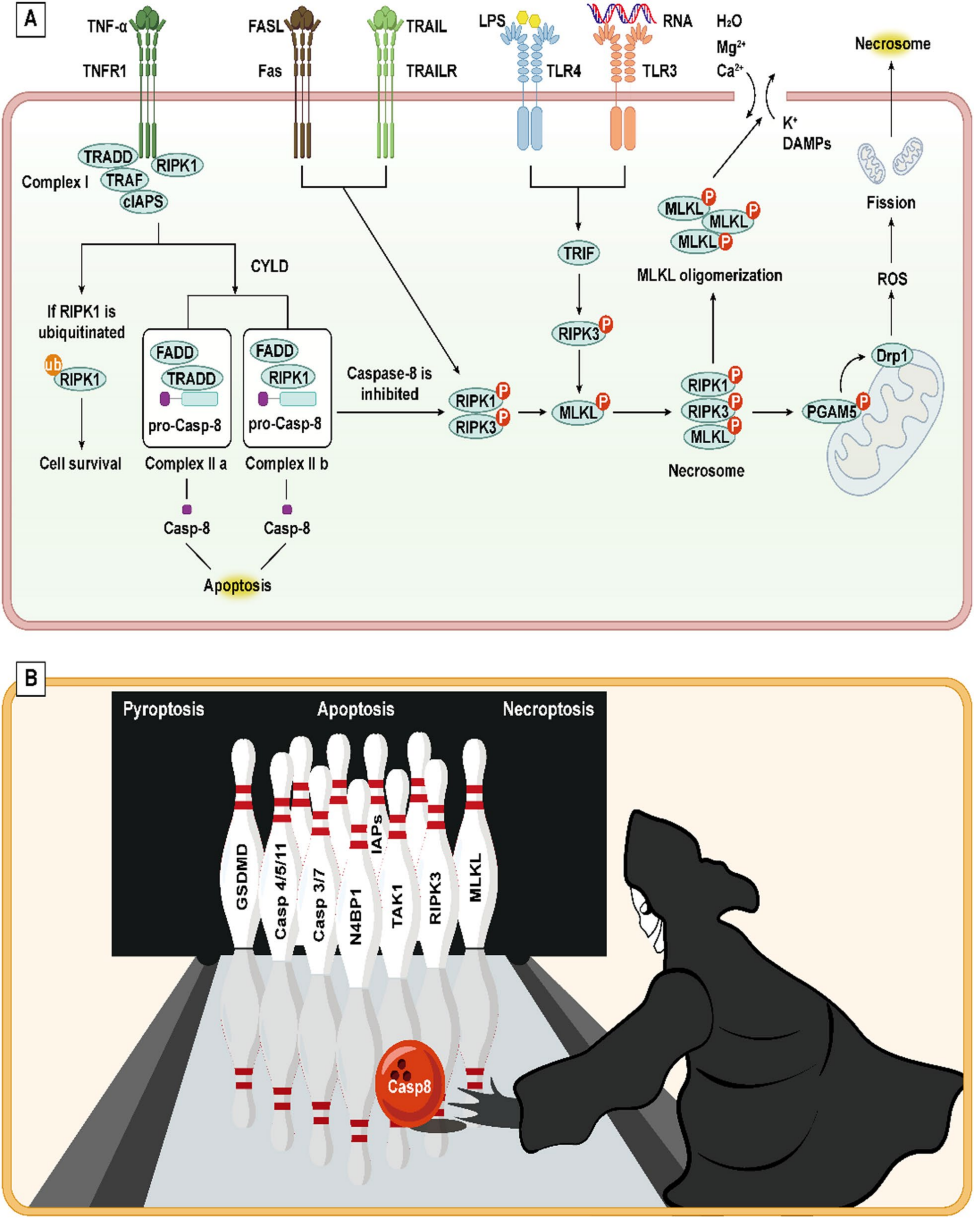
** A** The process of necrotic apoptosis. The activation of distinct death ligands by disparate external signalling stimuli prompts the phosphorylation of RIPK1, RIPK3, MLKL, and the formation of oligomeric complexes, which facilitate the rupture of the cell membrane and the release of cellular contents, resulting in the swelling and rupture of the cell and a peripheral inflammatory response. **B** Caspase-8 acts as a molecular switch that regulates the processes of apoptosis, pyroptosis, and necroptosis. The cell's fate is determined by the interactions of caspase-8 with both pro- and anti-apoptotic factors

In summary, there is often an antagonistic relationship between autophagy and necroptosis. The expression of MLKL can affect lysosomal integrity, thereby influencing autophagy. Guo et al*.* proposed that when cells were induced by oxidized low-density lipoprotein, MLKL overexpressed and activated mTOR pathway, down-regulated the expression of LC3-II and lysosomal associated membrane proteins to inhibit autophagy (Guo et al. 2019). In addition to the effects of MLKL, Liu et al. suggested that the natural compound shikonin induces RIP3 activation via ROS production in bladder cancer cells, which promotes necrotic apoptosis and targets the p62/Keap1 complex to impair autophagic flux (Liu et al. 2023a). However, in a study on retinal pigment epithelial cells, it was found that autophagy and necrotizing apoptosis promoted each other (Hwang et al. 2023). Therefore, the specific relationship between the two needs to be confirmed by further experiments. Regarding the relationship between ferroptosis and necroptosis, Müller et al. suggested that these two forms of cell death can be interconnected and even complementary. When one pathway is impaired, the other may compensate to ensure cell death (Müller et al. 2017) (Fig. 5).

### Necroptosis and DKD

Studies have shown that in HG-induced podocyte and in the glomeruli of DKD patients, there is a high positive expression of ubiquitin C-terminal hydrolase L1 (UCHL1), necroptosis signaling markers (RIPK1/3, MLKL), and apoptosis signaling markers (caspase-3). The specific mechanism involves UCHL1 promoting the apoptotic and necroptotic signaling cascades in HG-induced podocytes, with a particularly pronounced deubiquitination effect on RIPK1 and RIPK3. Consequently, under conditions where UCHL1 is regulated, necroptosis may have a more significant impact on HG-induced podocyte loss than apoptosis. Therefore, downregulating UCHL1 to inhibit the RIPK1/RIPK3/MLKL pathway could be a novel strategy for protecting podocytes (Xu et al. 2019). Qi et al. found that the RIPK1/RIPK3/MLKL immunostaining of RTECs in DKD patients confirmed the occurrence of necroptosis. The level of MLKL was positively correlated with lipid droplet accumulation and the degree of renal function deterioration in these patients. Animal studies have shown that the administration of a RIPK1 inhibitor effectively suppresses the activation of necroptosis in the renal tissues of DKD mice fed a HFD, reduces necroinflammation in the kidneys, and protects renal tissue (Yu et al. 2023). Sung found that in DKD mice, PINK1 deficiency not only led to increased ROS and mitochondrial dysfunction, but also led to increased levels of phosphorylation of RIPK1 and MLKL, which increased the onset of necroptosis, followed by more severe renal tubular injury and interstitial fibrosis (Sung et al. 2023). Yi et al*.* found that the expression of RIPK1, RIPK3 and p-p38MAPK increased when rats were treated with HG, and these effects could be cancelled by the application of adiponectin, which could significantly reduce the proteinuria level in DKD rats (Yi and OuYang 2019).

However, research on necroptosis in DKD is still developing, and several questions remain unresolved. While both podocytes and renal tubular cells are crucial in DKD progression, the specific responses of these cell types to different signaling pathways and the underlying mechanisms have not been thoroughly compared. For instance, Sung et al. demonstrated that overexpression of PINK1 in renal tubular cells inhibits necroptosis and activates mitophagy. It remains to be seen if a similar antagonistic relationship between necroptosis and autophagy exists in podocytes. Additionally, caspase-8 acts as a key regulator bridging apoptosis and necroptosis, but the effects of HG on caspase-8 activity are not well understood. Qi et al. found that HG and fatty acid stimulation lead to necroptosis in RTECs, but the precise intracellular signaling pathways involved are still unknown. In summary, further research is needed to elucidate the specific signaling pathways involved in necroptosis in DKD, particularly how these pathways interact with autophagy and apoptosis, and to determine how these mechanisms can be targeted for therapeutic benefit.

## The impact of NPs on DKD under different PCD modalities

### NPs' influence on apoptosis for DKD treatment

**Fig. 6:**
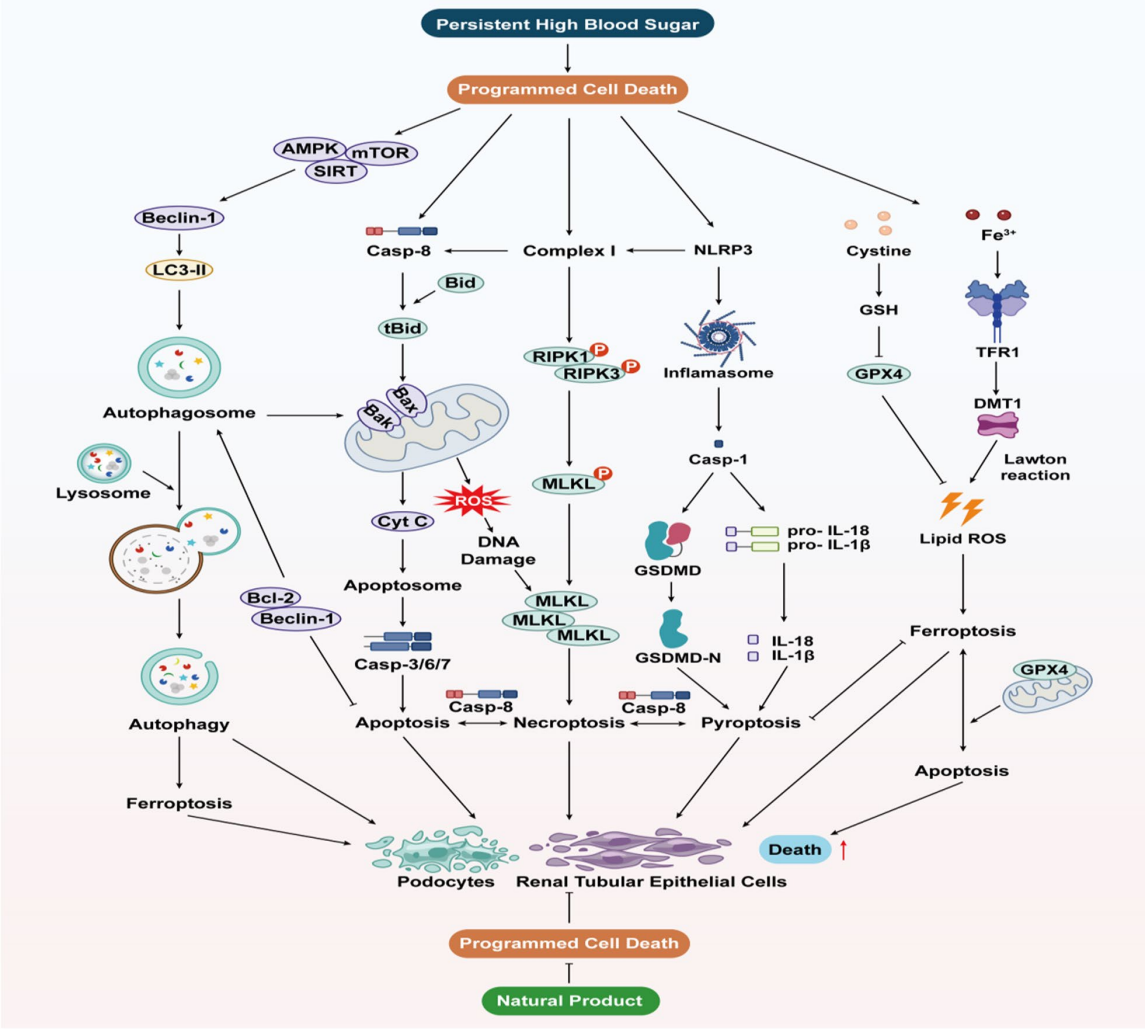
A sustained hyperglycaemic state has been demonstrated to promote programmed cell death and its corresponding downstream pathways, which collectively enhance autophagy, apoptosis, pyroptosis, necrotising apoptosis and ferroptosis. Crosstalk between the different modes of cell death has been observed to collectively promote renal cell death, including podocytes and renal tubular epithelial cells. Natural products have been shown to inhibit programmed cell death, thereby protecting the aforementioned cells

This section delves into the utilization of NPs for apoptosis targeting in the treatment of DKD. It primarily encompasses endogenous mitochondrial and ER apoptotic pathways, alongside the mitigation of inflammation and oxidative stress by NPs and their effect on apoptosis. The cell types involved include podocytes, renal tubular cells, and mesangial cells. Due to the multitude of drugs involved and the slight overlap of regulatory mechanisms and affected proteins, we have summarized the information into a table (Table 1), which is briefly discussed here (Fig. 6).

**Table 1:** NPs to target apoptosis as a means of combating DKD

Name	Sources	Structure	In vitro/In vivo	Model	Dose and Duration	Correlated target	Mechanisms	References
Z. officinale	Z. officinale	NA	In vivo	STZ induced Wistar rats	400, 800 mg/kg 6 weeks	Apoptosis, inflammation, oxidative stress	↓Cytc,caspase-3 ↓TNF-α, IL-1β ↑SOD,GSH,CAT ↓BUN, SCr	Al Hroob et al. (2018)
HSYA	Carthamus tinctorius L		In vivo	STZ induced Wistar rats	120 mg/kg 8 weeks	Apoptosis, inflammation, oxidative stress	↓Bax ↑Bcl-2 ↓caspase-3, ROS ↑SOD ↓TNF-α ↓FFA, LDH	Lee et al. (2020)
PN	Phylanthus niruri	NA	In vivo	STZ induced Wistar rats	200, 400 mg/kg 28 days	Apoptosis, inflammation, oxidative stress, fibrosis	↓Bax ↑Bcl-2 ↓caspase-3/9 ↓ TNF-α, IL-1β ↑SOD, GSH, CAT ↓VEGF, TGF-β	Giribabu et al. (2017)
Mangiferin	Mangifera indica		In vivo	STZ induced Wistar rats	40 mg/kg 4 weeks	Mitochondrial dependent apoptotic pathways	↓caspase-8, tBid ↓PARP ↓Bax, Cyt C ↓caspase-3/9 ↑Bcl-2, MMP ↓NF-κB, TNF-α ↓TGF-β1, MAPK	Pal et al. (2014)
Quercetin	Bupleuri		In vitro	40 mM HG induced MPCs	10, 20, 40 μM 24 h	EGFR	↓Bax ↓caspase-3 ↑Bcl-2 ↓BUN, UACR ↑Renin	Liu et al. (2021c)
			In vivo	C57BL/KSJ db/db rats	50, 100, 150 mg/kg 8 weeks			
Emodin	Rhubarb		In vitro	30 mM HG induced MPCs	20, 40 μM 24 h	PERK/eIF2α/ATF4	↑GPR78 ↓PERK, eIF2α ↓CHOP, ATF4 ↓Bax ↑Bcl-2 ↓BUN, SCr	Tian et al. (2018)
			In vivo	C57BL/6J rats	40,80 mg/kg 8 weeks			
AS-IV	Astragalus		In vitro	palmitate induced MPCs	20, 40, 80 μM 12 h	SERCA/SERCA2b/ER/mitochondrion	↑SERCA ↑SERCA2b ↑GPR78, Bcl-2 ↓ATF6, IRE1α, ↓PERK, eIF2α ↓CHOP, Ca^2+^ ↓Bax, Cyt c	Guo et al. (2016)
			In vivo	db/db rats	2, 6, 18 mg/kg 8 weeks			
AS-IV	Astragalus		In vitro	HG induced MPCs	10, 20, 40 μM 1 h	TRPC6/Ca^2+^	↓TRPC6 ↓Ca^2+^ ↓ NFAT2, Bax	Yao et al. (2016)
AS-IV	Astragalus		In vitro	HG induced MPCs	3, 10, 20, 40, 80, 100 μM 24 h	Nrf2-ARE/TFAM	↑Nrf2-ARE/TFAM ↓Bax, Cyt c ↓caspase-3 ↓ROS ↑SOD, GSH-Px ↑Nrf2, HO-1 ↑PGC-1α, ETC	Shen et al. (2023)
			In vivo	STZ induced C57BL/6J rats	6 mg/kg 10 weeks			
Berberine	Coptis chinensis		In vitro	Palmitate induced MPCs	0.4 μM 12 days	Drp1	↓Drp1 ↑PGC-1α, Bcl-2 ↓Bax, Cyt c ↓caspase-3,ROS ↓MMP-9 ↑SOD	Qin et al. (2019)
			In vivo	db/db rats	300 mg/kg 8 weeks			
Rb1	Ginseng		In vitro	HG stimulated MPCs	10 μM 72 h	AR	↓AR ↓ROS ↓Bax, Cyt c ↓caspase-3/9 ↓BUN, SCr	He et al. (2022)
			In vivo	FVB rats	40 mg/kg 7 weeks			
Gastrodin	Gastrodia elata		In vitro	HG stimulated MPC5	0.1, 1, 10, 100 μM 24 h	AMPK/Nrf2	↑AMPK, Nrf2 ↓Bax ↑Bcl-2 ↓caspase-1/3/6/9 ↓TNF-α, NLRP3 ↓MDA ↑SOD	Huang et al. (2022d)
CCPs	Cordyceps cicadae	NA	In vivo	db/db rats	75, 150, 300 mg/kg 12 weeks	miR-30a-3p/TRIM16	↑miR-30a-3p ↓TRIM16, ROS ↓Bax, caspase-3 ↑Bcl-2, SOD ↓TNF-α, IL-1β	Zheng et al. (2024)
AS-IV	Astragalus		In vitro	HG induced MPCs	10, 20, 40 μM 4 h	Klotho/FoxO1	↑Klotho/FoxO1 ↓Bax, caspase-3 ↑Bcl-2 ↓ROS ↑SOD ↑Podocin ↑Nephrin	Xing et al. (2021)
			In vivo	db/db rats	2, 6, 18 mg/kg 8 weeks			
Hirudin	Leeches		In vivo	STZ induced SD rats	Injected with 5 U 8 weeks	p38 MAPK/NF-κB	↓p38 MAPK ↓NF-κB ↓TNF-α, IL-1β	Han et al. (2020)
AS-IV	Astragalus		In vivo	STZ induced SD rats	5 mg/kg 12 weeks	miR-378/TRAF5	↑miR-378 ↓TRAF5 ↓caspase-3	LeiZhangRen et al. (2018)
AS-IV	Astragalus		In vivo	STZ induced SD rats	5 mg/kg 12 weeks	lncRNA-TUG1/TRAF5	↑lncRNA- TUG1 ↓TRAF5 ↓caspase-3	LeiZhangLi et al. (2018)
FMN	Astragalus membranaceus		In vitro	HG induced HK-2 cells	10, 30 μM 48 h	SIRT1/PGC-1α	↑SIRT1/PGC-1α ↓Bax, caspase-3 ↑Bcl-2 ↓Drp1, Fis1 ↑Mfn2, MMP ↓ROS	Huang et al. (2022e)
			In vivo	STZ induced SD rats	20 mg/kg 8 weeks			
GSPE	Grape seed	NA	In vivo	db/db rats	30 mg/kg 12 weeks	TXNIP p38MAP/ERK1/2	↓TXNIP, Cyt c ↓Bax, caspase-3 ↑Bcl-2,p38MAPK ↑ERK1/2	Wei et al. (2018)
C3G	Folium mori		In vitro	HG induced HK-2 cells	50 μM 48 h	TXNIP p38MAP/ERK1/2	↓TXNIP, Cyt c ↓Bax, caspase-3 ↑Bcl-2,p38MAPK ↑ERK1/2 ↓ROS ↑MMP	Wei et al. (2018)
NGR1	Panax notoginseng		In vitro	AGEs induced HK-2 cells	25 μM 24 h	Nrf2/HO-1	↑Nrf2/HO-1 ↓TGF-β ↓Bax ↑Bcl-2 ↓caspase-3/9 ↓ROS ↑MMP	Zhang et al. (2019)
			In vivo	db/db rats	30 mg/kg 20 weeks			
AS-IV	Astragalus		In vitro	HG induced HK-2 cells	10, 20, 40 μM 24 h	Nrf2/ARE	↑Nrf2/ARE ↓Bax ↑Bcl-2 ↓caspase-3/9 ↑SOD, GSH-Px ↓LPO, ROS	Wang and Guo (2019)
Salidroside	Rhodiola rosea		In vitro	HG induced HK-2 cells	100 μM 48 h	Bim	↓Bim ↓Bax ↑Bcl-2 ↓caspase-3 ↓ROS ↓BUN,SCr	Guo et al. (2018)
			In vivo	STZ induced Wistar rats	70 mg/kg 8 weeks			
Osthole	Cnidium monnieri		In vitro	HG induced HBZY-1 cells	1, 5, 10 μM 24 h	TGF-β1/Smads	↓TGF-β1/Smads ↓α-SMA, ROS ↑SOD, GSH-Px ↓NF-κB, TNF-α ↓IL-6, IL-1β ↓Apoptosis ↓BUN, SCr	Li et al. (2024)
			In vivo	STZ induced SD rats	25, 50, 100 mg/kg 8 weeks			
PCS	Psoralea corylifolia L	NA	In vivo	STZ induced C57BL/6 rats	500 mg/kg 8 weeks	Apoptosis/renal fibrosis	↓ PARP, Bad ↓TGF-β1, PAI-1 ↓ROS	Seo et al. (2017)

The rhizome extract of Zingiber officinale (Z. officinale), Hydroxysafflor Yellow A (HSYA) from Carthamus tinctorius L., P. niruri leaves aqueous extract (PN), and mangiferin have demonstrated efficacy in reducing inflammation and oxidative stress to inhibit apoptosis in DKD rats. They exert renal protection by reducing blood urea nitrogen (BUN), serum creatinine (SCr), and urine protein levels (Al Hroob et al. 2018; Giribabu et al. 2017; Lee et al. 2020; Pal et al. 2014). PN and mangiferin additionally inhibit renal fibrosis in DKD rats, evidenced by the down-regulation of vascular endothelial growth factor (VEGF) and collagen, respectively. Mangiferin further inhibits the mitochondrial apoptotic pathway by suppressing TNF-α/caspase-8/Bid (Pal et al. 2014).

#### Regulation of podocyte apoptosis by NPs

Quercetin can decrease the expression of Bax and caspase-3 in HG-induced MPCs and db/db mice by reducing epidermal growth factor receptor (EGFR) expression and increasing Bcl-2 expression, thereby protecting podocytes (Liu et al. 2021c). Emodin has been shown in in vivo and in vitro experiments to reduce GRP78 expression and HG-induced MPC apoptosis, increase cell viability, and upregulate podocyte marker renin expression by inhibiting the PERK/eIF2α/ATF4 signaling pathway(Tian et al. 2018).

Astragaloside IV (AS-IV) modulates podocyte apoptosis by impacting ER and mitochondrial homeostasis. Guo et al. confirmed that abnormal expression of Sarco/endoplasmic reticulum Ca^2+^-ATPase (SERCA) could lead to Ca^2+^ imbalance, induce ERS, and subsequently trigger endogenous mitochondrial apoptosis. AS-IV ameliorates SERCA and SERCA2 expression, reverses the aforementioned process, decreases the expression of GRP78, PERK, CHOP, and Cyt C, restores podocyte activity, and alleviates diabetic renal injury (Guo et al. 2016). Yao et al. found that AS-IV inhibits HG-induced transient receptor potential channel 6 (TRPC6) in MPCs, reducing intracellular Ca^2+^ concentration, and also inhibits Bax and nuclear factor of activated T cells (NFAT2) expression, collectively down-regulating podocyte apoptosis (Yao et al. 2016). Shen et al. discovered that HG stimulation reduces the mitochondria-specific electron transport chain (ETC) complex of podocytes, impairs mitochondrial biogenesis, and increases podocyte apoptosis. AS-IV activates the Nrf2-antioxidant response element (ARE)/mitochondrial transcription factor A (TFAM) signaling pathway, counteracts the aforementioned trend, and reduces mitochondrial dysfunction to protect podocytes (Shen et al. 2023). Berberine from Coptis chinensis inhibits matrix metalloproteinase-9 (MMP-9) expression, a podocyte injury marker in DKD mice, and promotes SD expression. Berberine achieves this by inhibiting Drp1 expression, reducing mitochondrial fission, and increasing peroxisome proliferator-activated receptor gamma coactivator 1-alpha (PGC-1α) expression, thereby inhibiting podocyte apoptosis (Qin et al. 2019). Ginsenoside Rb1 (Rb1) binds to Aldose reductase (AR), mitigates AR-induced mitochondrial damage in HG-cultured podocytes, reduces ROS, Cyt C, and caspase-9 expression, and restores MMP to protect podocytes (He et al. 2022).

Gastrodin and cordyceps cicadae polypeptides (CCPs) inhibit apoptosis and protect diabetic kidney tissue by suppressing podocyte inflammation and oxidative stress. Specifically, Gastrodin activates the AMPK/Nrf2 pathway in MPC5 cells to exert a protective effect (Huang et al. 2022d). CCPs induce the miR-30a-3p/TRIM16 pathway to reverse EMT of podocytes (Zheng et al. 2024). Additionally, AS-IV regulates the Klotho/FoxO1 pathway to suppress oxidative stress and attenuate podocyte apoptosis in DKD (Xing et al. 2021). Inhibition of the p38 MAPK/NF-κB pathway by Hirudin suppresses inflammation and macrophage infiltration, alleviates podocyte apoptosis, and reduces BUN, SCr, and proteinuria levels in DKD rats (Han et al. 2020). Lei et al. confirmed that AS-IV down-regulates TRAF5 expression to inhibit podocyte apoptosis in DKD rats and reduce BUN, Scr, and proteinuria levels in rats. Subsequent studies have proposed that AS-IV increases miR-378 and lncRNA-TUG1 expression, thereby inhibiting TRAF5 and achieving the aforementioned renal protection (LeiZhangRen et al. 2018; LeiZhangLi et al. 2018).

#### Regulation of renal tubular cells and mesangial cell apoptosis by NPs

Formononetin (FMN) from Astragalus membranaceus upregulates Sirt1/PGC-1α pathway, inhibits the expression of mitochondrion fission proteins DRP1 and FIS1 in HK-2 cells induced by HG, increases mitochondrial fusion protein Mfn2 expression, reduces ROS accumulation, restores MMP, and mitigates renal tubular cell apoptosis (Huang et al. 2022e). Grape seed procyanidin (GSPE) and cyanidin-3-*O*-β-glucoside chloride (C3G) are additional extracts of Anthocyanin. In vivo, GSPE inhibits TXNIP activity and increases antioxidant enzyme p38 MAPK and extracellular signal-regulated kinase 1/2 (ERK1/2) activity to reduce renal tubular cell apoptosis. Similar conclusions were drawn by C3G in vitro using HG-induced HK-2 cells (Wei et al. 2018). Notoginsenoside R1 (NGR1) promotes Nrf2/HO-1 expression to inhibit oxidative stress, reduce apoptosis of HK-2 cells, and mitigate renal fibrosis caused by TGF-β (Zhang et al. 2019). In HG-induced HK-2 cells, AS-IV modulates the Nrf2/ARE pathway to enhance antioxidant enzyme activities, such as SOD, GSH, and catalase (CAT), thereby reducing apoptosis (Wang and Guo 2019) Salidroside downregulates the expression of pro-apoptotic proteins Bim and Bax, thereby reducing apoptosis in renal tubular cells of DKD rats and HG-induced HK-2 cells (Guo et al. 2018).

In HG-cultured mesangial cells (HBZY-1 cells), Osthole reduces TGF-β1/Smads expression, inhibits inflammatory factors and α-SMA protein levels (a direct reflection of renal fibrosis degree), increases SOD and GSH levels, and subsequently reduces apoptosis, thus alleviating glomerular volume increase and mesangial matrix proliferation in DKD rats (Li et al. 2024). Psoralea corylifolia L. seed (PCS) reduces fibrosis marker gene and pro-apoptotic gene PARP expression, and diminishes fibrosis and apoptosis of mesangial cells in DKD rats (Seo et al. 2017).

### NPs' Influence on autophagy for DKD treatment

This section explores the process of using NPs to target autophagy in the treatment of DKD, mainly involving nutrition perception pathways and mitochondrial-induced autophagy pathways, as well as the effects of NPs on autophagy and apoptosis crosstalk. Due to the large number of drugs involved and the slight overlap of regulatory mechanisms and affected proteins, we make it into a table (Table 2), which is only briefly discussed here.

**Table 2:** NPs to target autophagy as a means of combating DKD

Name	Sources	Structure	In vitro/In vivo	Model	Dose and duration	Correlated target	Mechanisms	References
Kaempferol	Broccoli		In vivo	db/db mice	50, 100 mg/kg 12 weeks	AMPK/mTOR	↑AMPK ↓mTOR ↑Beclin1, LC3II ↓p62 ↑Bcl-2 ↓Bax, caspase-3	Sheng et al. (2022)
Emodin	Rhubarb		In vivo	STZ inducing SD rats	20, 40 mg/kg 8 weeks	AMPK/mTOR	↑AMPK ↓mTOR ↑Beclin1, LC3II/I ↓p62 ↓Bax, caspase-3	Liu et al. (2021b)
Berberine	Coptis chinensis		In vitro	HG induced MPCs	2.5 μM 20 h	AMPK/mTOR	↑AMPK ↓mTOR ↑Beclin1, LC3II/I ↓p62, caspase-3 ↑Podocin ↑Nephrin	Jin et al. (2017)
Geniposide	Gardenia		In vivo	STZ inducing C57BL/6J rats	50 mg/kg 5 weeks	AMPK/AKT/ULK1	↑AMPK ↓AKT ↑ULK1 ↑Beclin1, LC3II/I ↓TNF-α, IL-6 ↓PARP, caspase-3	Dusabimana et al. (2021)
Mangiferin	Mangifera indica		In vivo	STZ inducing SD rats	12.5, 25, 50 mg/kg 12 weeks	AMPK/mTOR	↑AMPK ↓mTOR ↑p-ULK1 ↑Beclin1, LC3II ↓p62	Wang et al. (2018)
Isoorientin	Fenugreek		In vitro	HG induced MPC5 cells	40 μM 72 h	PI3K/AKT/TSC2/mTOR	↓PI3K/AKT ↓TSC2 ↓mTOR ↑Beclin1, LC3II/I ↓p62	Kong et al. (2023)
			In vivo	STZ induced C57BL/6J rats	10, 20, 40 mg/kg 2 months			
NGR1	Panax notoginseng		In vitro	HG induced MPC5 cells	20 μM 24 h	PI3K/AKT/mTOR	↓PI3K/AKT ↓mTOR ↑Beclin1, LC3II ↓caspase-3/9 ↓Bax, PARP ↑Bcl-2, Bcl-xL ↑podocin ↑nephrin	Huang et al. (2017a)
Curcumin	Turmeric		In vitro	MPC5 cells	40 μM 24 h	PI3K/AKT/mTOR	↓PI3K/AKT ↓mTOR ↓TWIST1 ↑Beclin1, LC3 ↓p62	Tu et al. (2019)
			In vivo	STZ induced SD rats	300 mg/kg 8 weeks			
Curcumin	Turmeric		In vitro	HG induced MPC5 cells	20, 40, 80 μM 48, 78 h	Beclin1/UVRAG/Bcl-2	↑Beclin1, LC3 ↑UVRAG, Atg5 ↓p62 ↑Bcl-2 ↓Bax, caspase-3	Zhang et al. (2020)
			In vivo	STZ induced SD rats	200 mg/kg 8 weeks			
Catalpol	Rehmannia glutinosa		In vitro	HG induced MPC5 cells	1, 5, 10 μM 48 h	mTOR/TFEB	↓mTOR ↑TFEB nuclear translocation ↑mRFP ↑LC3B ↓p62 ↓RhoA, Cdc42	Chen et al. (2019b)
			In vivo	STZ induced C57BL/6J rats	30, 60, 120 mg/kg 8 weeks			
Berberine	Coptis chinensis		In vitro	HG induced MPC5 cells	30, 60, 90 μM 24 h	mTOR/P70S6K/4EBP1	↑mTOR ↑P70S6K/4EBP1 ↑LC3II/I ↓p62 ↓caspase-3	Li et al. (2020a)
Tripterygium glycoside	Tripterygium	NA	In vitro	11 nM HG induced MPC5 cells	1.25 μM 24 h	mTOR/Twist1	↓mTOR/Twist1 ↑Beclin1, LC3II/I ↓EMT, E-cadherin ↓Apoptosis	Tao et al. (2021)
Tripterygium glycoside	Tripterygium	NA	In vitro	MPC5 cells	1.25 μM 24 h	β-arrestin-1	↓β-arrestin-1 ↑LC3II/I ↓p62 ↓Apoptosis	Zhan et al. (2019)
Hispidulin	Plantago asiatica		In vitro	HG induced MPC5 cells	2, 5 μM 24 h	Pim1/p21/mTOR	↓Pim1/p21 ↓mTOR ↑Beclin1 ↓Apoptosis	Wu et al. (2018)
Puerarin	Radix puerariae		In vivo	STZ induced C57BL/6J rats	5, 10, 20, 40 mg/kg 12 weeks	HMOX-1, SIRT1/AMPK	↑HMOX-1 ↑SIRT1/AMPK ↓LKB1 acetylation ↑Beclin1, LC3II/I ↓Apoptosis	Li et al. (2020c)
AS-IV	Astragalus		In vitro	HG induced MPC5 cells	20, 50, 100 μM 48 h	SIRT1/NF-κB p65	↑SIRT1 ↓p65 acetylation ↑Beclin1, LC3II ↓ECM, α-SMA ↓E/N-cadherin	Wang et al. (2019)
			In vivo	Polygenic KK-Ay mice	40 mg/kg 12 weeks			
Puerarin	Radix puerariae		In vivo	STZ induced C57BL/6J rats	40, 80 mg/kg 8 weeks	PERK/eIF2α/ATF4	↑PERK/eIF2α ↑ATF4 ↑Beclin1, LC3II/I ↑Atg5 ↓p62 ↓BUN, Scr	Xu et al. (2020b)
AS-IV	Astragalus		In vitro	HG induced MPC5 cells	80 μM 72 h	SERCA2b, AMPK/mTOR	↑SERCA2b ↑AMPK/mTOR ↑Beclin1, LC3II/I ↓ATF6, PERK, ↓IRE1α, p-eIF2α ↓GRP78, CHOP ↓caspase-3/12	Guo et al. (2017)
			In vivo	STZ induced C57BL/6J rats	3, 6, 12 mg/kg 8 weeks			
Wogonin	Scutellaria baicalensis Georgi		In vitro	HG induced MPC5 cells	4, 8, 16 μM 24 h	Bcl-2/Beclin1	↑Bcl-2 ↑Bcl-2/Bax ↑Beclin1, LC3 ↑Atg7 ↓p62 ↓Bax, caspase-3 ↓TNFα, IL-6 ↑podocin ↑nephrin	Liu et al. (2022b)
			In vivo	STZ induced C57BL/6J rats	10, 20, 40 mg/kg 12 weeks			
Resveratrol	Grape leaves		In vitro	HG induced Human podocytes	0, 5, 10, 15 μM 48 h	miR-383-5p	↓miR-383-5p ↑Beclin-1, LC3II ↓p62 ↑Atg5 ↓Bax, caspase-3	Huang et al. (2017b)
			In vivo	db/db mice	10 mg/kg 12 weeks			
Celastrol	Tripterygium		In vitro	HG induced Mouse podocytes	0.1, 0.2, 0.6, 1.0, 1.5, 2 μM 5 h	HO-1	↑HO-1 ↑Beclin-1, LC3II/I ↓p62 ↓ROS ↓TNF-α, IL-6 ↓Apoptosis	Zhan et al. (2018)
DHM	Ampelopsis Michx		In vitro	HG induced NRK-52E cells	1 μM 24 h, 48 h	miR-155-5p/PTEN PI3K/AKT/mTOR	↓miR-155-5p ↑PTEN ↓AKT/mTOR ↑Beclin-1, LC3II/I ↓p62 ↓RIF, α-SMA ↓Col IV, FN	Guo et al. (2020)
			In vivo	STZ induced SD rats	100 mg/kg 10 weeks			
Tretinoin	Tripterygium		In vitro	HG induced HMCs	10 µM 48 h	miR-141-3p/PTEN/Akt/mTOR	↓miR-141-3p ↑PTEN ↓Akt/mTOR ↑LC3 ↓p62 ↓RIF, ECM ↓Col IV, FN	Li et al. (2017)
			In vivo	STZ induced SD rats	200 μg/kg 12 weeks			
Asiatic acid	Cyclocarya paliurus		In vitro	HG/TGF-β1 induced HK-2 cells	1, 5 μM 24 h	TGF-β1/smad3	↓TGF-β1/TGF-β RI ↓smad3 phosphorylated ↑LC3, LAMP1 ↓p62 ↓ α-SMA ↓EMT, E-cadherin	Zhang et al. (2022b)
			In vivo	STZ induced SD rats	10, 30 mg/kg 15 weeks			
Resveratrol	Grape leaves		In vivo	STZ induced SD rats	5 mg/kg 4 months	SIRT1	↑SIRT1 ↑Beclin-1, LC3II/I ↓p62 ↑Atg5,7 ↓TNF-α,IL-1β/6/10	Ma et al. (2016)
Syringic acid	Many plants		In vitro	HG induced NRK-52E cells	10, 20 μM 48 h	Nrf2	↑Nrf2, NQO-1 ↑Beclin-1, LC3 ↑Atg3/5/7 ↓p62 ↓ROS, MDA ↑SOD, GSH ↓BUN, SCr	Sherkhane et al. (2023)
			In vivo	STZ induced SD rats	20, 50 mg/kg 4 weeks			
Astilbin	Glabrous greenbrier rhizome		In vitro	HG induced HK-2 cells	10, 20 μM 24 h	PI3K/AKT	↑PI3K/AKT ↓Beclin-1, LC3II/I ↑p62 ↑Bcl-2 ↓Bax, caspase-3	ChenSun et al. (2018)
Diosgenin	Rhizoma dioscoreae		In vivo	STZ induced SD rats	20 mg/kg 8 weeks	PINK1/Parkin, Drp1/Mfn2, AMPK/mTOR, PERK/eIF2α/ATF4	↑PINK1/Parkin ↑AMPK/mTOR ↑Beclin-1, LC3 ↓Drp1 ↑Mfn2 ↓PERK/eIF2α ↓CHOP, Cyt C ↓Bax, caspase-9/12 ↓TNF-α, IL-6/1β ↑SOD ↓MDA	ZhongLiu et al. (2022)
Diosgenin	Rhizoma dioscoreae		In vitro	HG induced HK-2 cells	1, 2, 4 μM 24 h	CaMKK2, PINK1/Parkin, AMPK/mTOR	↑CaMKK2, ↑PINK1/Parkin ↑AMPK/mTOR ↑Beclin-1, LC3 ↓Drp1 ↑Mfn1/2 ↓Bax, caspase-12	Zhong et al. (2023)
			In vivo	STZ induced SD rats	10, 20 mg/kg 8 weeks			
Jujuboside A	Jujube	NA	In vivo	STZ induced SD rats	20 mg/kg 8 weeks	CaMKK2, PINK1/Parkin, AMPK/mTOR, PERK/eIF2α/ATF4	↑CaMKK2, ↑PINK1/Parkin ↑AMPK/mTOR ↓PERK/eIF2α ↓CHOP, Cyt C ↑Beclin-1, LC3 ↓Bax, caspase-9 ↑SOD ↓MDA	ZhongLuo et al. (2022)
Isorhamnetin	Hippophae rhamnoides L		In vivo	STZ induced Wistar rats	50 mg/kg 4, 8 weeks	miR-15b, miR-34a, miR-633	↓miR-15b ↓miR-34a ↓miR-633 ↑ULK1, WIPI ↑FYCO1, TECPR ↑LC3II/I ↓p62	Matboli et al. (2021)

#### NPs' impact on podocyte autophagy

Kaempferol, emodin, berberine, geniposide, and mangiferin have been demonstrated to promote AMPK and inhibit mTOR, thus activating podocyte autophagy, inhibiting apoptosis, alleviating podocyte foot process fusion and mesangial expansion, and protecting renal tissue from diabetic injury (Dusabimana et al. 2021; Jin et al. 2017; Liu et al. 2021b; Sheng et al. 2022; Wang et al. 2018). Interestingly, geniposide further inhibits the AKT/mTOR pathway, leading to ULK1 activation and the promotion of autophagy, while simultaneously reducing oxidative stress and inflammation associated with macrophage infiltration (Dusabimana et al. 2021).

Isoorientin, NGR1, and Curcumin inhibit the PI3K/AKT/mTOR pathway to stimulate autophagy to protect podocytes. Besides stimulating autophagy, Isoorientin regulates mitochondrial homeostasis and reduces apoptosis through mitochondrial autophagy (Kong et al. 2023). NGR1 inhibits the expression of the apoptotic protein and gene PARP, increases the expression of podocyte markers podocin and nephrin, and promotes the restoration of the cytoskeleton (Huang et al. 2017a). Curcumin reduces EMT occurrence and E-cadherin and TWIST1 protein expression in MPC5 cells (Tu et al. 2019). Zhang et al. also demonstrated that Curcumin regulates Beclin1/UVRAG/Bcl-2, increases autophagy, and inhibits podocyte apoptosis (Zhang et al. 2020).

Catalpol, berberine, and tripterygium glycoside inhibit mTOR in HG-induced podocytes, promoting downstream transcription factor EB (TFEB) nuclear translocation, P70 ribosomal protein S6 kinase (P70S6K)/eukaryotic translation initiation factor 4E-binding protein 1 (4EBP1) activities, and inhibiting downstream Twist1 to exert pro-autophagic effects. Catalpol culture results in reduced RhoA and Cdc42 expression, reducing cytoskeletal migration and enhancing cytoskeletal stability (Chen et al. 2019b). The addition of Berberine can inhibit podocyte apoptosis (Li et al. 2020a). Tripterygium glycoside treatment reduces EMT and apoptosis (Tao et al. 2021). Furthermore, tripterygium glycoside can stimulate autophagy and suppress β-arrestin-1 to alleviate podocyte apoptosis (Zhan et al. 2019). Conversely, hispidulin inhibits Pim1/p21/mTOR to promote autophagy, inhibit apoptosis (Wu et al. 2018).

Puerarin and AS-IV upregulate SIRT1 to activate autophagy or participate in ERS regulation to protect podocytes. In Li et al.'s study, puerarin affected HMOX-1 and SIRT1/AMPK pathways, decreased liver kinase B1 (LKB1) acetylation, and exerted an anti-apoptotic role (Li et al. 2020c). Wang et al. confirmed that AS-IV upregulated SIRT1 expression, downregulated NF-κB subunit p65 acetylation, activated autophagy, and reduced EMT (Wang et al. 2019). Xu et al. employed puerarin to affect the PERK/eIF2α/ATF4 pathway, increase autophagy, and decrease ERS, alleviating clinical indexes in DKD rats (Xu et al. 2020b). Guo et al. found that AS-IV affected SERCA2b to inhibit ERS, simultaneously upregulated AMPK to promote autophagy while mitigating apoptosis in podocytes (Guo et al. 2017).

In addition to the above NPs, there are some NPs that regulate other autophagy factors to treat DKD. Wogonin upregulates Bcl-2, disrupts Bcl-2/Beclin1 binding, releases Beclin1, and mediates HG-induced autophagy and apoptosis crosstalk in MPC5 cells, reducing inflammation, apoptosis, and promoting autophagy to decelerate DKD progression (Liu et al. 2022b). Resveratrol inhibits miR-383-5p expression, promoting autophagy, and delaying apoptosis in podocytes (Huang et al. 2017b). Celastrol can promote HO-1 expression, reduce ROS production and inflammatory factor levels, improve autophagy activity, and thus reduce apoptosis (Zhan et al. 2018).

#### Regulation of renal tubular cell autophagy by NPs

Some NPs modulate renal tubular cell autophagy and reduce renal fibrosis to treat DKD. Phosphatase and tensin homolog (PTEN) controls cell adhesion and angiogenesis and inhibits Akt/mTOR pathway activity. MicroRNA targets PTEN to reduce fibrosis and enhance autophagy. Dihydromyricetin (DHM) down-regulates miR-155-5p in NRK-52E cells on HG-induced rat renal tubular epithelium, promoting PTEN expression, inhibiting PI3K/AKT/mTOR pathway activity, stimulating autophagy, and reducing renal intermittent fibrosis (RIF) caused by collagen IV (Col IV) and fibronectin (FN) accumulation (Guo et al. 2020). Tretinoin downregulates miR-141-3p in human mesangial cells (HMC) induced by HG, activates PTEN/Akt/mTOR pathway, restores autophagy, and reduces renal fibrosis (Li et al. 2017). Asiatic acid binds to TGF-β type I receptor (TGF-βRI), inhibiting downstream Smad3 activation, upregulating the autophagy-lysosome system, down-regulating fibrotic protein expression, and delaying EMT and RIF (Zhang et al. 2022b).

Resveratrol upregulates SIRT1 expression, enhances autophagy, and reduces inflammation, ameliorating kidney injury in DKD rats. Additionally, resveratrol induces HK-2 cells to improve autophagy activity and inhibit apoptosis under hypoxia (Ma et al. 2016). Syringic acid increases autophagy-related protein and antioxidant-related protein expression in DKD rats and NRK-52E cells, such as Nrf2 and NQO-1, attenuating DKD progression (Sherkhane et al. 2023). Considering the optimal range of autophagy activity, astilbin activates the PI3K/Akt pathway in HK-2 cells under HG culture, reducing autophagy, alleviating apoptosis, and protecting cells (ChenSun et al. 2018).

#### Regulation of mitophagy and other autophagy pathways by NPs

Diosgenin modulates mitochondrial quality through PINK1/PARKIN and Drp1/Mfn2, regulates ER homeostasis through PERK/eIF2α/ATF4, and regulates AMPK/mTOR pathway to activate autophagy, thus inhibiting mitochondrial disorder, apoptosis, inflammation, and oxidative stress caused by ER (ZhongLiu et al. 2022). Subsequently, the team proposed that CaMKK2 mediates PINK1/PARKIN to restore mitochondrial autophagy and, as an upstream of AMPK, restores autophagy. Diosgenin targets and regulates CaMKK2 in DKD rats and HG-induced HK-2 cells to achieve the aforementioned process, while regulating Drp1 and Mfn2 to improve mitochondrial dynamics (Zhong et al. 2023). The mechanism of Jujuboside A in treating DKD is similar to Diosgenin targeting CaMKK2 in the aforementioned experiments (ZhongLuo et al. 2022), demonstrating the importance of autophagy, mitochondrial autophagy, and ER homeostasis in DKD treatment.

Moreover, isorhamnetin regulates autophagy epigenetic regulatory factors miR-15b, miR-34a, and miR-633 to target downstream autophagy transcription signals ULK1, WIPI, FYCO1, and TECPR mRNA expression, promoting autophagy and kidney tissue protection (Matboli et al. 2021).

### NPs' impact on ferroptosis for DKD treatment

This section elucidates the utilization of NPs in targeting ferroptosis for the treatment of DKD. The discussion primarily encompasses the involvement of GPX4, system XC-, iron ion-related proteins, antioxidant Nrf2, and the effects of NPs on ferroptosis, apoptosis, and autophagy crosstalk. Given the multitude of drugs involved and the slight overlap in regulatory mechanisms and affecting proteins, a summarized table (Table 3) is provided, with brief discussion presented herein.

**Table 3:** NPs to target ferroptosis as a means of combating DKD

Name	Sources	Structure	In vitro/in vivo	Model	Dose and duration	Correlated target	Mechanisms	References
Ginkgolide B	Ginkgo		In vitro	HG induced MPCs	20, 40, 80 μM 24 h	GPX4 ubiquitination	↓GPX4 ubiquitination ↑GPX4, FTH1 ↓TFR1 ↓ROS, α-SMA ↓Total cholesterol ↓Triglyceride	Chen et al. (2022)
			In vivo	Male C57BL/KsJ db/db mice	100, 200 mg/kg 3 months			
MGM	Mangifera indica	NA	In vivo	STZ induced SD rats	200 mg/kg 4 weeks	FSP1/CoQ10 Gpx4, MAPK/NF-κB, p-PI3K/p-AKT	↑FSP1/CoQ10 ↑GPX4 ↓ROS, MDA ↓MAPK/NF-κB ↓JNK/p38 MAPK ↓TNF-α、IL-1β/6 ↑p-PI3K/p-AKT ↓Insulin resistance	Zhao et al. (2023a)
Puerarin	Pueraria lobata		In vitro	HG induced HBZY-1 cells	1, 10 μM 24 h	Ferroptosis and ECM	↑SLC7A11, SLC3A2 ↑GPX4 ↓ACSL4 ↓TFR1 ↑FTH1 ↓ROS, MDA ↓α-SMA, FN, TGF-β ↓BUN, SCr	Hou et al. (2024)
			In vivo	STZ induced SD rats	100 mg/kg 8 weeks			
Emodin	Rhubarb		In vitro	HG induced HK-2 cells	40 μM 48 h	Nrf2	↑Nrf2 ↑SLC7A11 ↑GPX4 ↓ACSL4 ↓TFR1 ↑FTH1 ↓ROS, MDA	Ji et al. (2023)
			In vivo	STZ induced SD rats	100 mg/kg 12 weeks			
Quercetin	Bupleuri		In vitro	HG induced HK-2 cells	25 μM 48 h	Nrf2	↑Nrf2 ↑GPX4 ↑GSH ↓ACSL4 ↓ROS, MDA	Zhang et al. (2024a)
			In vivo	STZ induced SD rats	100 mg/kg 12 weeks			
Quercetin	Bupleuri		In vitro	HG induced HK-2cells	6.25, 12.5, 25, 50, 100 μM 48 h	Nrf2/HO-1	↑Nrf2/HO-1 ↑GPX4, SLC7A11 ↑GSH ↓ACSL4 ↓TFR1 ↑FTH1 ↓ROS, MDA	Feng et al. (2023)
			In vivo	Male C57BL/KsJ db/db mice	25, 100 mg/kg 12 weeks			
UMB	umbelliferae plants		In vitro	HG induced HK-2 cells	5 μM 24 h	Nrf2/HO-1	↑Nrf2/HO-1 ↑GPX4, GSH ↓ACSL4 ↑ΔΨm ↓ROS, MDA ↓BUN, SCr	Jin and Chen (2022)
			In vivo	C57BL/KsJ db/db mice	20 mg/kg 4 weeks			
Platycodin D	Platycodon grandiflorum		In vitro	HG induced HK-2 cells	1, 2.5, 5 μM 24 h	GPX4	↑GPX4,SLC7A11 ↓ACSL4, Fe^2+^ ↓TFR1 ↑FTH1 ↓ROS, MDA, LDH	Huang et al. (2022c)
Vitexin	Passion flower		In vitro	HG induced HK-2 cells	10, 20 μM 24 h	GPX4	↑GPX4, SLC7A11 ↑GSH ↓Fe^2+^ ↓ROS, MDA, LDH ↓Col I, TGF-β1	Zhang et al. (2023a)
			In vivo	STZ induced SD rats	10, 20 mg/kg 4 weeks			
Glabridin	Licorice		In vivo	STZ induced SD rats	50 mg/kg 3 weeks	VEGF/Akt/ERK	↓VEGF/Akt/ERK ↑GPX4 ↑SLC7A11, SLC3A2 ↓TFR1 ↑CAT, GSH, SOD	Tan et al. (2022)
Chicoric acid	Cichoric		In vitro	HG induced NRK-52E cells	5, 10, 20 μM 24 h	PAQR3, PI3K/AKT/GPX4	↑PAQR3 Ubiquitination ↓PAQR3 ↓P110α ↑PI3K/AKT/GPX4 ↑GSH	Zhang et al. (2024b)
			In vivo	STZ induced SD rats	7.5, 15, 30 mg/kg 6 weeks			
Tanshinone IIA	Salvia miltiorrhiza Bunge		In vitro	HG induced MPC5 cells	10 μM 24 h	ELAVL1/ACSL4	↓ELAVL1, ACSL4 ↑GPX4 ↓ROS, MDA ↓TNF-α、IL-1β/6 ↓Bax ↑Bcl-2	Zhu et al. (2024)
RLP	Rosa laevigata Michx	NA	In vivo	STZ induced C57BJ/6 rats	20, 40, 80 mg/kg 4 weeks	GPX4, PI3K/AKT	↑GPX4 ↑PI3K/AKT ↑SOD,GSH ↓MDA ↓TNF-α、IL-1β/6 ↓Bax ↑Bcl-2 ↓caspase-3/9	Zhang et al. (2023b)
Calycosin	Astragali Radix		In vitro	HG induced HK-2 cells	10, 20, 40 μM 24 h	GPX4	↑GPX4 ↑GSH ↓NCOA4 ↓ROS,MDA,LDH ↓BUN,SCr	Huang et al. (2022b)
			In vivo	db/db rats	10, 20 mg/kg 4 weeks			
Germacrone	Rhizoma curcuma		In vitro	HG induced HK-2 cells	50 μM 72 h	mtDNA/cGAS/STING, PINK1/Parkin	↓mtDNA/cGAS/STING ↑PINK1/Parkin ↑LC3II/I ↑GPX4, FTH1 ↓ROS, MDA	Wang et al. (2023b)
			In vivo	C57BL/KsJ db/db mice	10, 30, 40, 60 mg/kg 4 weeks			

#### Regulation of ferroptosis in podocytes and mesangial cells by NPs

Ginkgolide B exhibits inhibitory effects on GPX4 ubiquitination, enhances FTH1 expression, and reduces TFR1 expression in DKD mice and HG-induced MPC5 cells, thereby inhibiting ferroptosis and reducing ROS and α-SMA expression (Chen et al. 2022). Mangiferin monosodium salt (MGM) augments GPX4 and FSP1/CoQ10 axis expression, suppresses lipid drivers promoting renal prolapse mediated by ACSL4, mitigates ROS and lipid accumulation, and inhibits ferroptosis in DKD rats. Additionally, MGM attenuates MAPK/NF-κB and activates p-IRS1(Tyr608)/p-PI3K/p-Akt, thereby ameliorating HG-induced podocyte inflammation and insulin resistance (Zhao et al. 2023a).

In DKD rats and HG-damaged HBZY-1 cells, Puerarin not only mitigates ECM accumulation and fibrosis but also acts on GPX4, SLC7A11, and SLC3A2 to restrain lipid accumulation. Furthermore, Puerarin enhances ferroportin FTH1 expression while inhibiting transferrin TFR1 expression, thereby regulating iron storage to inhibit ferroptosis and enhance cell viability (Hou et al. 2024).

#### Regulation of ferroptosis in renal tubular cells by NPs

Nrf2, a crucial transcription factor in antioxidative stress regulation, mitigates HG-induced cellular ferroptosis. Emodin activates Nrf2, enhancing the antioxidant activity of HK-2 cells under HG injury. Similar to Puerarin, Emodin regulates the system Xc-, GPX4, and iron ion-related storage transporters to collectively inhibit ferroptosis (Ji et al. 2023). Quercetin activation of Nrf2 in HK-2 cells increases GSH and GPX4 expression, thereby reducing renal injury driven by oxidative stress and ferroptosis (Zhang et al. 2024a). Additionally, other experiments have demonstrated that quercetin, by activating Nrf2, subsequently promotes the activation of downstream HO-1, thereby exerting antioxidant effects. This process regulates lipid metabolism and iron storage, inhibits ferroptosis and oxidative stress, and protects HG-induced HK-2 cells (Feng et al. 2023). Coincidentally, umbelliferone (UMB) activates the Nrf2/HO-1 pathway to inhibit ferroptosis and ROS accumulation, restoring MMP and ameliorating kidney injury in DKD rats (Jin and Chen 2022).

In HG-induced HK-2 cells, NPs targets to elevate GPX4 and decrease lactate dehydrogenase (LDH), MDA and lipid ROS expression to inhibit ferroptosis and antioxidant, which is also an important pathway for the treatment of DKD. The use of Platycodin D can also inhibit the levels of TFR1 and ACSL4, and up-regulate the expression of FTH1 and SLC7A11 to jointly inhibit ferroptosis (Huang et al. 2022c). After Vitexin treatment, in addition to the above effects, it can also increase the expression of SLC7A11 and inhibit fibrosis (Col I, TGF-β1) to jointly improve cell viability (Zhang et al. 2023a).

In studys utilizing the renal tubular epithelium of rats under HG injury (NRK-52E). Glabridin downregulates the VEGF/Akt/ERK pathway, promoting GPX4, system Xc-, SOD, and CAT expression, protecting renal cells by inhibiting ferroptosis and oxidative stress (Tan et al. 2022). Chicoric acid enhances progestin and ADIPO Q acceptor 3 (PAQR3) ubiquitination, activating the PI3K/AKT/GPX4 pathway, inhibiting ferroptosis, and treating DKD (Zhang et al. 2024b).

#### Crosstalk between ferroptosis and other PCDs regulated by NPs

In the crosstalk between ferroptosis and apoptosis, ELAVL1 interacts with ACSL4. Tanshinone IIA regulates ELAVL1 and inhibits ACSL4 expression, thus inhibiting ferroptosis, inflammation, and apoptosis of MPC5 cells induced by HG (Zhu et al. 2024). Rosa laevigata Michx. polysaccharide (RLP) modulates tryptophan metabolism in DKD mic, activating GPX4 and PI3K/AKT pathways, inhibiting ferroptosis and apoptosis, and exerting anti-inflammatory and antioxidant effects to protect damaged kidney tissue (Zhang et al. 2023b).

In the crosstalk between ferroptosis and autophagy, Calycosin downregulates NCOA4 expression, reducing ferritin autophagy, and upregulates GPX4 expression, inhibiting LDH, MDA, ROS, ferroptosis, and antioxidation to alleviate kidney injury caused by high glucose (Huang et al. 2022b). Germacrone inhibits the mtDNA/Cyclic GMP-AMP synthase (cGAS)/stimulator of interferon genes (STING) pathway in HG-2 cells induced by HG. Additionally, it regulates the PINK1/Parkin pathway, promoting mitophagy, improving ferroptosis in HK-2 cells, inhibiting inflammation and oxidative stress, and therapeutically treating DKD (Wang et al. 2023b).

### NPs' impact on pyroptosis for DKD treatment

This section explores the use of NPs to target pyroptosis for the treatment of DKD, mainly involving the NLRP3 inflammasome and some antioxidant factors such as TXNIP, Nrf2 and HO-1, as well as the influence of the crosstalk between pyroptosis and ferroptosis, autophagy nutrient sensing pathways under the influence of NPs. Because of the large number of drugs involved and the slight overlap in regulatory mechanisms and affected proteins, we make it into a table (Table 4), which is only briefly discussed here.

**Table 4:** NPs to target pyroptosis as a means of combating DKD

Name	Sources	Structure	In vitro/in vivo	Model	Dose and duration	Correlated target	Mechanisms	References
Tanshinone IIA	Salvia miltiorrhiza Bunge		In vitro	HG induced HRGEC	20 μM 24 h	TXNIP/NLRP3	↓TXNIP ↑Trx1 ↓NLRP3 ↓GSDMD-N ↓IL-1β, caspase-1	Wu et al. (2023)
			In vivo	db/db mice	2 ml/kg 12 weeks			
Calycosin	Astragali Radix		In vivo	STZ induced SD rats	5, 10 mg/kg 10 weeks	TXNIP/NLRP3, NF-κB	↓TXNIP/NLRP3 ↓NF-κB, Nrf2 ↓MDA, NO ↓IL-10	Yosri et al. (2022)
Salidroside	Rhodiola rosea		In vitro	HG induced HBZY-1 cells	20,40 μM 24 h	TXNIP/NLRP3	↓TXNIP/NLRP3 ↓ASC, caspase-1 ↓ROS, MDA ↑SOD ↓ECM ↓Col IV,FN	Wang et al. (2017a)
Punicalagin	Pomegranate polyphenols		In vivo	STZ induced C57BJ/6 rats	20 mg/kg 8 weeks	TXNIP/NLRP3, NOX4	↓TXNIP/NLRP3 ↓NOX4, GSDMD ↓IL-1β, caspase-1 ↓BUN, UACR	An et al. (2020)
Ginsenoside compound K	Black ginseng		In vitro	HG induced HBZY-1 cells	10, 20, 40 μM 48, 72 h	TXNIP/NLRP3, NOX1/4, NF-κB p65/P38 MAPK	↓TXNIP/NLRP3 ↓ASC, caspase-1 ↓NOX1/4 ↓NF-κB/P38MAPK ↓TNF-α, IL-1β/18 ↓ROS, MDA ↑SOD, GSH-PX ↓BUN, SCr	Song et al. (2018a)
			In vivo	STZ induced C57BJ/6 rats	10, 20, 40 mg/kg 8 weeks			
Ginsenoside Rg5	Black ginseng	NA	In vivo	STZ induced C57BJ/6 rats	30, 60 mg/kg 6 weeks	TXNIP/NLRP3, NOX4, NF-κB p65/P38 MAPK	↓TXNIP/NLRP3 ↓ASC, caspase-1 ↓NOX4 ↓NF-κB/P38MAPK ↓IL-1β, IL-18 ↓ROS, MDA ↑SOD, GSH-PX	Zhu et al. (2020)
AS-IV	Astragalus		In vitro	HG induced MPC5 cells	50, 75, 100 μM 12 h	Klotho/NF-κB/NLRP3	↑Klotho ↓NF-κB ↓NLRP3 ↓ROS ↓ASC, caspase-1 ↓IL-1β, IL-18 ↓GSDMD-N ↑ΔΨm, nephrin	He et al. (2023)
			In vivo	STZ induced SD rats	40, 80 mg/kg 12 weeks			
Triptolide	Tripterygium wilfordii		In vitro	HG induced MPC5 cells	10 μM 48 h	Nrf2/HO-1, ROS, NLRP3	↑Nrf2/HO-1 ↓ROS, MDA ↓NLRP3 ↓ASC, caspase-1 ↓IL-1β, IL-18 ↑SOD, GSH-PX ↓BUN, SCr, FBG ↑podocin ↑nephrin	Lv et al. (2023a)
			In vivo	STZ induced C57BL/6J rats	100 μg/kg 12 weeks			
Solasonine	Solanum melongena		In vitro	HG induced MPC5 cells	5, 10, 20 μM 48 h	Nrf2/NLRP3	↑Nrf2 ↓ROS, MDA ↓NLRP3 ↓ASC, caspase-1 ↓IL-1β, IL-18 ↑SOD, CAT ↑podocin ↑nephrin	Zhang et al. (2022a)
Catalpol	Rehmannia glutinosa		In vitro	HG induced MPC5 cells	1, 5, 10 μM 48 h	AMPK/SIRT1/NF-κB	↑AMPK/SIRT1 ↓NF-κB ↓NLRP3 ↓IL-1β ↓ASC, caspase-1 ↓ROS, MDA ↑SOD, GSH-PX ↓BUN, SCr	Chen et al. (2020)
			In vivo	STZ induced C57/B6 rats	100, 150, 200 mg/kg 4 weeks			
Geniposide	Gardenia jasminoides		In vivo	STZ induced C57/B6 rats	25, 50 mg/kg 4 weeks	AMPK/SIRT1/NF-κB	↑AMPK/SIRT1 ↓NF-κB ↓NLRP3 ↓GSDMD-N ↓ASC, caspase-1 ↓TNF-α, IL-1β/18 ↓ROS, MDA ↑SOD, GSH-PX ↓BUN, SCr, GBM	Li et al. (2020b)
Fucoidan	Laminaria japonica		In vitro	HG induced MPC5 cells	20 μM 24 h	AMPK/mTOR/NLRP3	↑AMPK ↓mTOR, NLRP3 ↓GSDMD-N ↓ASC, caspase-1 ↓IL-1β/6/18 ↓ColI, FN, ECM ↓TGF-β1, Smad2/3 ↑podocin ↑nephrin	Wang et al. (2022a)
			In vivo	STZ induced SD rats	120 mg/kg 4 weeks			
Ginsenoside Rg1	Black ginseng		In vitro	HG induced MPC5 cells	50 μM 48 h	mTOR/NF-κB/NLRP3	↓mTOR, NLRP3 ↓NLRP3 ↓ASC ↓caspase-1, IL-1 ↓UACR, BUN	Wang et al. (2020)
			In vivo	STZ induced SD rats	50 mg/kg 8 weeks			
TFA	Abelmoschus manihot	NA	In vitro	HG induced MPC5 cells	20 μM 24 h	METTL3, m^6^A PTEN/PI3K/Akt	↑PTEN/PI3K/Akt ↓NLRP3 ↓ASC, caspase-1 ↓GSDMD-N ↑nephrin ↑ZO-1,WT1	Liu et al. (2021a)
Tanshinone IIA	Salvia miltiorrhiza Bunge		In vitro	HG induced HK-2 cells	1, 5, 10 μM 24 h	TGF-β1	↓TGF-β1 ↑Nrf2 ↓GSDMD-N ↓TNF-α, ↓IL-1β/6/18 ↓ColI, FN	Li et al. (2022c)
Syringaresinol	Annona Montana		In vitro	HG induced HBZY-1 cells	50 μM 48 h	NLRP3, Nrf2/HO-1	↓NLRP3 ↓caspase-1 ↓GSDMD-N ↑Nrf2/HO-1 ↓ROS ↑SOD	Li et al. (2023a)
			In vivo	STZ induced C57BL/6J rats	25 mg/kg 8 weeks			
Curcumin	Curcuma longa		In vitro	HG induced HK-2 cells	5, 10, 15 μM 48 h	NLRP3/caspase-1/IL-1β/IL-18	↓NLRP3 ↓caspase-1 ↓IL-1β, IL-18 ↓Col IV, FN	Lu et al. (2017)
			In vivo	db/db mice	200 mg/kg 16 weeks			
Naringin	Grapefruit		In vitro	HG induced glomerular mesangial cells	5, 10, 20, 40, 80 μM 48 h	NLRP3/caspase-1/IL-1β/IL-18	↓NLRP3 ↓ASC ↓caspase-1 ↓IL-1β, IL-18	ChenWei et al. (2018)
DHQ	Larix sibirica Ledeb		In vitro	HG induced HBZY-1/HK-2 cells	10, 20, 40, 80 μM 48 h	NLRP3/caspase-1/IL-1β/IL-18	↓NLRP3 ↓caspase-1 ↓IL-1β, IL-18 ↓ROS ↓Col IV, FN	Ding et al. (2018)
			In vivo	STZ induced SD rats	25, 50 mg/kg 4 weeks			
Schisandrin A	Schisandra chinensis		In vitro	HG induced HRGEC	25, 50, 100 μM 48 h	AdipoR1/AMPK/ROS/mitochondrial damage	↑AdipoR1/AMPK ↓ROS, MDA ↑SOD, GSH-PX ↑Nrf2/HO-1 ↓TXNIP/NLRP3 ↓caspase-1, IL-1β ↓GSDMD-N ↑GPX4	X WangQ Li et al. (2022)
			In vivo	STZ induced C57BL/6 rats	25,50,100 mg/kg 8 weeks			

#### TXNIP and NOX4-mediated pyroptosis regulated by NPs

Oxidative stress can also regulate the occurrence of pyroptosis. Many experiments on the treatment of DKD rats by NPs have proved that TXNIP inhibits the occurrence of pyroptosis mediated by the activation of downstream NLRP3 inflammatory bodies when regulating oxidative stress. Tanshinone IIA regulates TXNIP/Thioredoxin1 (Trx1) in vivo to inhibit the activation of downstream NLRP3 and reduce the production of GSDMD-N to inhibit endothelial cell pyroptosis (Wu et al. 2023). In addition to inhibiting TXNIP, calycosin can also inhibit NF-κB and Nrf2 to further inhibit the occurrence of inflammation and oxidative stress and restore damaged kidney structure (Yosri et al. 2022). Salidroside inhibits pyroptosis in HG-induced HBZY-1 cells by suppressing the TXNIP/NLRP3 signaling pathway. Additionally, it mitigates oxidative stress in glomerular mesangial cells and downregulates the expression of ECM-related proteins Col IV and FN, thereby reducing ECM accumulation (Wang et al. 2017a).

NOX4, as an oxidase, can play an antioxidant role in the body. Punicalagin acts on both TXNIP and NOX4 to effectively attenuate mitochondrial damage and alleviate DKD-related manifestations (An et al. 2020). Ginsenoside compound K and Ginsenoside Rg5 share the same mechanism, regulating TXNIP and NOX4 in DKD mice, and inhibiting the phosphorylation of NF-κB p65 and p38 MAPK to further alleviate endothelial cell pyroptosis and delay the progress of DKD (Song et al. 2018a; Zhu et al. 2020).

#### Regulation of pyroptosis in podocytes by NPs

AS-IV increases the expression of Klotho, thus inhibiting NF-κB/NLRP3 pathway, restoring MMP and protecting podocytes exposed to HG (He et al. 2023). Triptolide stimulates Nrf2/HO-1 pathway to alleviate oxidative stress injury and prevent the activation of NLRP3 inflammatory corpuscles, thus protecting MPC5 cells and DKD rats induced by HG (Lv et al. 2023a). Like Triptolide, solasonine also activates Nrf2 to impede downstream ROS and NLRP3 activation, inhibiting cellular oxidative stress, pyroptosis and apoptosis (Zhang et al. 2022a).

Some NPs can target the nutritional sensing pathway in autophagy to play an anti-pyroptosis role in podocytes. Both Catalpol and Geniposide can phosphorylate the AMPK/SIRT1 pathway and inhibit the expression of downstream NF-κB, thereby blocking oxidative stress and inflammatory response with NLRP3-mediated podocyte pyroptosis, and improving renal structural and functional abnormalities (Chen et al. 2020; Li et al. 2020b). In the in vitro and in vivo experiments of DKD, Fucoidan has excellent ability to regulate AMPK/mTOR pathway, which can be targeted to inhibit the accumulation of ECM and GBM to alleviate renal fibrosis, and also inhibit the activation of NLRP3 and reduce podocyte pyroptosis (Wang et al. 2022a). Ginsenoside Rg1 inhibits the mTOR/NF-κB pathway in podocytes under HG conditions, blocks the activation of NLRP3 to not cause pyroptosis, and improves the renal function of DKD mice (Wang et al. 2020). Total Flavones of Abelmoschus manihot (TFA) plays an anti-pyroptosis role in HG-induced MPC5 cells, which requires the participation of Mettle 3-dependent M6 A modification, so as to up-regulate PTEN and activating PI3K/Akt, thus inhibiting the assembly and activation of downstream NLRP3 inflammatory corpuscles (Liu et al. 2021a). Unfortunately, although the above experiments involved autophagy-regulated targets, none of them explicitly mentioned the specific role of autophagy and pyroptosis crosstalk in the protection of podocytes, so further studies are needed.

#### Regulation of pyroptosis in renal tubular cells and mesangial cells by NPs

Tanshinone IIA inhibits the expression of ECM and Nrf2 related genes through TGF-β1 pathway, and it is proved that Tanshinone IIA plays a protective role in cells by inhibiting pyroptosis rather than apoptosis (Li et al. 2022c). Syringaresinol inhibits the NLRP3/Caspase1/GSDMD pyroptosis pathway on the one hand, and on the other hand promotes the Nrf2 nuclear translocation to activate downstream HO-1 to produce antioxidation to jointly treat kidney injury in DKD mice (Li et al. 2023a).

Curcumin acts on HK-2 cells, Naringin acts on mesangial cells, and Dihydroquercetin (DHQ) acts on both cells. All three can inhibit the occurrence of pyroptosis in HG-induced cells mediated by the NLRP3/caspase-1/IL-1β/IL-18 pathway, and increase cell viability for the treatment of DKD (ChenWei et al. 2018; Ding et al. 2018; Lu et al. 2017).

#### Pyroptosis and ferroptosis regulated by NPs

In DKD rats and Human Renal Glomerular Endothelial Cells (HRGEC) induced by HG, Schisandrin A targets AdipoR1 protein, activates downstream AMPK, and reduces mitochondrial damage induced by lipid ROS accumulation and oxidative damage of cells. Subsequently, Schisandrin A resists the occurrence of ferroptosis through Nrf2/HO-1/GPX4 on the one hand, and inhibits the TXNIP/NLRP3 pathway on the other hand, so as to play a role in treating DKD (X WangQ Li et al. 2022).

### NPs' impact on necroptosis for DKD treatment

This section explores the use of NPs to target necroptosis for the treatment of DKD, mainly involving RIPK3 and MLKL protein expression, the effects of pyroptosis and autophagy crosstalk under the influence of NPs. Details are shown in Table 5.

**Table 5:** NPs to target necroptosis as a means of combating DKD

Name	Sources	Structure	In vitro/in vivo	Model	Dose and duration	Correlated target	Mechanisms	References
Paeoniflorin	Paeonia lactiflora		In vitro	HG induced MPC5 cells	40,80,160 μM 24 h	TNFR1, RIPK1/RIPK3/MLKL	↑TNFR1 ubiquitination ↓TNFR1 ↓RIPK1/RIPK3 ↓MLKL ↓TNF-α, IL-1β ↓BUN, SCr, GBM	X WangXQ Liu et al. (2022)
			In vivo	STZ induced C57BL/6J rats	100 mg/kg 6 weeks			
Curcumin	Curcuma longa		In vitro	HG induced MPC5 cells	50,100,150,200 μM 24 h	RIPK3	↓RIPK3 ↓MLKL ↓ROS ↓VEGF, TGF-β ↑nephrin	Chung et al. (2022)
CALE	Cassia auriculata	NA	In vitro	HG induced RGE cells	50,100,150 μM 72 h	LC3-II, RIPK1/3, p38MAPK	↓LC3-II ↓RIPK1/RIPK3 ↓p38MAPK ↓BUN, SCr, FBG	Al Shahrani et al. (2021)
			In vivo	STZ induced SD rats	150 mg/kg 10 weeks			

Paeoniflorin directly binds to TNFR1 protein and induces TNFR1 ubiquitination, which in turn regulates the RIPK1/RIPK3 pathway, inhibits MLKL expression and suppresses necroptosis. In addition, Paeoniflorin ameliorates inflammation and GBM thickening to reduce cellular damage and combat DKD (X WangXQ Liu et al. 2022). Curcumin has anti-oxidation effect, which leads to the decrease of ROS and RIPK3, and then hinders the activation of VEGF, TGF-β and MLKL, reduces the necroptosis, oxidative stress injury and fibrosis of podocytes (Chung et al. 2022). Cassia auricula ethyl leaf extract (CALE) can improve the dysfunction and structural abnormality of kidney injury in DKD rats by inhibiting autophagy and necrotizing apoptosis pathways, which is demonstrated by the reduction of LC3-II, RIPK1/3 and p38MAPK (Al Shahrani et al. 2021).

## New treatment methods based on administration route and cell intervention

### Nanotechnology—a new choice of drug delivery system

When DKD occurs, renal glomerular filtration and tubular secretion are impaired, and even increasing the drug concentration does not adequately ensure that target cells receive the required amount of drug (Rawat et al. 2023). Despite the demonstrated therapeutic efficacy of NPs in DKD, traditional administration routes pose challenges such as the first-pass effect of the liver, action of digestive enzymes in the gastrointestinal tract, and gastric acid, all of which diminish bioavailability and therapeutic outcomes. In recent years, the integration of nanotechnology into medicine has emerged as a promising solution. Nanomaterials of various sizes, predominantly liposomes, polymers, nanomicelles, and nanoparticles, can be conjugated with NPs to cater to the diverse biomolecule reabsorption needs of glomeruli and tubules. This integration enhances the targeted delivery and distribution capabilities of drugs, regulates release profiles, and augments therapeutic efficacy (Rawat et al. 2023; Yuan et al. 2023). For instance, Ahangarpour et al. developed solid lipid nanoparticles (SLN) of myricitrin through cold homogenization, demonstrating superior efficacy of SLN over myricitrin alone in inhibiting TGF-β, NF-κB, and renal apoptosis in DKD mice (Ahangarpour et al. 2019). Tong et al. observed more significant changes in clinical indicators of DKD rats treated with a Quercetin nanoparticle complex compared to free quercetin, possibly due to delayed release and increased in vivo circulation time of quercetin conferred by the nanoparticle complex (Tong et al. 2017). Additionally, Wu et al. developed a single-molecule nano-conjugate comprising methylprednisolone and carrier albumin, enabling direct drug delivery to cultured human podocytes via glomerular filtration for therapeutic intervention (Wu et al. 2017). Thus, the amalgamation of NPs with nanotechnology holds promise in targeting PCDs for the treatment of DKD.

### Cell therapy—a new direction for cell death

For renal cell death in DKD, MSC may provide new ideas for the treatment of DKD. MSC, with the ability to repair, can differentiate into various cell types when stimulated, such as glomerular endothelial cells (Habiba et al. 2024). Notably, MSCs exhibit a unique behavior termed non-apoptotic membrane blistering, enabling them to traverse the kidney's barrier by leveraging the matrix metalloproteinases family's functions, ultimately facilitating cell regeneration at the site (Habiba et al. 2024). At the same time, MSC-derived exosomes can also up-regulate autophagy or promote macrophage polarization to inhibit the formation of renal fibrosis and other cellular pathological reactions in DKD (Ebrahim et al. 2018; Zhang et al. 2022c). In a randomized controlled trial in Europe, it was shown that the cell therapy of intravenous injection of Anti-CD 362 Antibody-Selected Allogeneic MSCs (ORBCEL-M) for DKD patients was more safe and well tolerated. And compared with placebo, the decline rate of eGFR in ORBCEL-M group was more obvious within 18 months(Perico et al. 2023). In a clinical trial in Australia, it was also concluded that intravenous injection of allogeneic Mesenchymal Precursor Cells in DKD patients had a tendency to stabilize or improve eGFR and mGFR at the 12th week, which confirmed the effectiveness of MSC in the treatment of DKD (Packham et al. 2016). Interestingly, according to the latest research, MSCs can be combined with nanotechnology to treat kidney injury in DKD mice. Wang et al. developed Fe_3_O_4_ coated polyphosphate nanoparticle-internalized placeholder-MSCs (PL-MSCs), which can enhance the magnetic targeted therapy ability of PL-MSCs, improve the homing of DKD mice to renal tissue, and exert anti-inflammatory and anti-fibrosis properties (Wang et al. 2024b).

## Limitations

All forms of cell death present promising therapeutic targets that could play a significant role in correcting the progression of DKD in the future. However, the regulatory mechanisms of PCD in the onset and development of DKD remain poorly understood. Beyond the deficiencies mentioned earlier, we also posit that PCD may overlap within the same stage of the disease but contribute differently to DKD progression at various stages. How the types and roles of PCD evolve during different stages of DKD progression, how different PCDs interact in kidney damage in DKD, and the exploration of potential biomarkers related to PCD in the course of the disease—all warrant more detailed systematic investigation. Further elucidation of the relationship between cell–cell communication and PCD, as well as the interplay between epigenetics and PCD, will aid in a better understanding of the mechanisms by which PCD operates in DKD pathogenesis.

Currently, research on NPs targeting of PCD for the treatment of DKD is primarily based on in vitro and in vivo experiments, with a dearth of high-quality clinical studies to confirm their clinical efficacy. Additionally, the design of some experiments necessitates a more rigorous and reliable approach.Taking GFR as an example, GFR is often closely associated with the occurrence of proteinuria. In animal studies, indicators such as BUN and serum SCr are typically measured to assess GFR. Although it was mentioned earlier that NPs targeting PCDs can reduce the elevated levels of BUN and SCr in disease states, further clinical validation is still lacking. It should also be noted that the protective effect of NPs on GFR is limited; although they can reduce levels of BUN and SCr indicators, these levels typically remain higher than those in the normal control group of rats after treatment. Only a few studies, such as that by Sherkhane et al., have mentioned that high concentrations of Syringic acid can restore BUN and SCr to normal levels (Sherkhane et al. 2023). Interestingly, while most NPs significantly reduce both BUN and SCr, a minority may preferentially lower one of the indicators. The varying degrees of GFR recovery by the aforementioned NPs may also be influenced by factors such as the severity of DKD, the type of medication, and the dosage.

However, it is imperative to consider the limitations of traditional drug delivery routes, which can restrict the direct application of NPs in animal experiments and clinical settings. The bioavailability of NPs is influenced by various factors within the body, leading to suboptimal levels that cannot meet the unique demands of the kidney for concentrated and potent drugs. Therefore, there is a need to enhance the development of NPs, improving their absorption and targeting capabilities, and reducing their metabolic rates to precisely modulate PCDs. Novel drug delivery pathways that leverage nanotechnology, utilizing nanocarriers of varying sizes and materials, are gaining attention as they can fulfill the aforementioned requirements. Additionally, new cell therapies, primarily based on MSCs, can compensate for the tissue void left by cellular death, and existing clinical studies have demonstrated efficacy in patients with DKD. The unclear pharmacological mechanisms and poor pharmacokinetics of the drugs themselves are also significant factors affecting the progression of treatment.

Therefore, in future research, it will be essential to conduct more clinical trials to thoroughly assess the toxicological profile and long-term outcomes of these therapies, as well as to compare their efficacy with that of first-line clinical treatments. A focus on modulating the crosstalk between PCD pathways could harness the multi-target potential of NPs to its fullest extent. Moreover, leveraging advanced technologies and innovative approaches to address the inherent limitations of these drugs will be crucial in enhancing their efficacy and reliability.

## Conclusions and prospects

DKD is a diabetic microvascular complication characterized by a series of complex metabolic disorders under persistent hyperglycemic conditions. Once it progresses to ESRD, treatment often becomes more challenging than for other kidney diseases. Therefore, timely prevention and management of DKD are crucial for improving the long-term quality of life for patients. The pathogenesis of DKD is multifaceted, primarily driven by metabolic disturbances induced by hyperglycemia, which ultimately impairs the morphology and function of renal cells, including podocytes and RTECs, thereby exacerbating disease progression. PCD, orchestrated by a series of well-coordinated gene expression events, plays a pivotal role in eliminating damaged cells in response to various internal and external stimuli. By targeting and regulating different modes of cell death, PCD effectively mitigates the pathophysiological consequences of metabolic imbalances in vivo, thereby restoring glomerular filtration and renal tubular reabsorption function and maintaining renal tissue homeostasis.

Among the various PCD mechanisms, normal apoptosis of podocytes and RTECs represents a physiological outcome of cellular metabolism. However, excessive apoptosis can result in podocyte detachment from the GBM, compromising the integrity of the glomerular filtration barrier and leading to proteinuria. Moreover, there exists a significant correlation between the extent of tubulointerstitial injury and long-term renal function. While moderate autophagy facilitates the efficient recycling of cellular components and energy within renal cells, excessive autophagic activity can exacerbate cellular demise. The disturbance of glycolipid metabolism, induced by HG, impairs the production of antioxidants and disrupts mitochondrial homeostasis. This, contributes to the excessive intracellular accumulation of LPO and iron ions in kidney cells, which ultimately results in ferroptosis. Additionally, pyroptosis and necroptosis, characterized by their pro-inflammatory nature, trigger cell death concomitant with an inflammatory cascade in the surrounding tissues, exacerbating the destabilization of renal tissue.

Most studies have traditionally focused on investigating individual PCD pathways, operating under the assumption that these pathways function independently and in parallel. However, emerging evidence suggests that different forms of PCD exhibit intricate crosstalk dynamics, both temporally and spatially. Moreover, it has become increasingly apparent that a cell death pathway can not only augment other pathways but also suppress the expression of alternative death pathways. For instance, caspase-8 serves as a pivotal regulator that controls apoptosis, pyroptosis, and necroptosis, blurring the boundaries between the inflammatory consequences of pyroptosis or necroptosis and the immunosilencing characteristics of apoptotic cell death. Additionally, the Bcl-2/Beclin1 complexes mediate a bidirectional influence between apoptosis and autophagy, exerting both promotive and inhibitory effects on these processes. Autophagy modulates the occurrence of ferroptosis through mechanisms such as ferritinophagy, mitophagy, and lipophagy. Similarly, a reciprocal relationship exists between pyroptosis and autophagy, mediated by the interplay between NLRP3 inflammasome and autophagy. Moreover, an antagonistic interaction between pyroptosis and ferroptosis has been elucidated. Additionally, the necroptosis effector MLKL influences autophagy by affecting lysosomal integrity, while necroptosis and ferroptosis mutually potentiate each other, exacerbating cellular sensitivity to both processes.

As a natural drug source, NPs has the characteristics of low cost and high safety, and its multi-target characteristics are the advantage of NPs in regulating different PCD to treat DKD. For example, a single NPs can regulate different PCD crosstalk: Kaempferol, Diosgenin, Emodin, NGR1, Curcumin, etc.can promote autophagy and inhibit apoptosis to exert anti-DKD effects. RLP, Schisandrin A, Germacrone and Calycosin in NPs that inhibit ferroptosis can inhibit apoptosis and pyroptosis, respectively, and promote mitophagy and ferritinophagy. CALE can inhibit autophagy and necroptosis. In addition to their effects on different PCDs, NPs can target various pathways associated with the same or different PCDs to elicit therapeutic effects. For example, AS-IV targets different antioxidant, anti-inflammatory factors, and nutrient sensing pathways to prevent apoptosis, pyroptosis, and activate autophagy. Curcumin can enhance podocyte autophagy and inhibit HK-2 cell pyroptosis, while Tanshinone IIA exhibits anti-pyroptotic and anti-ferroptotic properties. Moreover, the multi-target pathway of NPs extends to the regulation of cellular pathological processes such as oxidative stress and inflammation, contributing to their effectiveness in DKD treatment.

The specific role of PCD in the pathogenesis of DKD remains an area that requires further investigation. Moreover, NPs are currently challenged by low bioavailability, poorly understood pharmacological mechanisms, and inadequate pharmacokinetics, which are compounded by a lack of extensive clinical research. Future studies should delve deeper into the interrelationships and dominant roles of various forms of PCD in DKD to elucidate the multi-cellular, multi-factorial regulatory mechanisms of cell death during the disease process. Additionally, enhancing the bioavailability of NPs and exploring drugs that can target and modulate multiple types of PCD are essential. Leveraging nanotechnology and cell therapy as entry points, it is crucial to actively seek new drug delivery pathways and treatment protocols, and to expedite clinical trials to better and more efficiently serve the broad population of DKD patients.

## Data Availability

No datasets were generated or analysed during the current study.
